# Characterizing metabolic drivers of *Clostridioides difficile* infection with activity-based hydrazine probes

**DOI:** 10.3389/fphar.2023.1074619

**Published:** 2023-01-26

**Authors:** Katelyn A. Bustin, Arwa Abbas, Xie Wang, Michael C. Abt, Joseph P. Zackular, Megan L. Matthews

**Affiliations:** ^1^ Department of Chemistry, University of Pennsylvania, Philadelphia, PA, United States; ^2^ Division of Protective Immunity, Children’s Hospital of Pennsylvania, Philadelphia, PA, United States; ^3^ Department of Microbiology, Perelman School of Medicine, University of Pennsylvania, Philadelphia, PA, United States; ^4^ Department of Pathology and Laboratory Medicine, Perelman School of Medicine, University of Pennsylvania, Philadelphia, PA, United States

**Keywords:** cofactor, ABPP, *Clostridioides difficile* infection, druggable modality, Stickland fermentation, hydrazine pharmacophore

## Abstract

Many enzymes require post-translational modifications or cofactor machinery for primary function. As these catalytically essential moieties are highly regulated, they act as dual sensors and chemical handles for context-dependent metabolic activity. *Clostridioides difficile* is a major nosocomial pathogen that infects the colon. Energy generating metabolism, particularly through amino acid Stickland fermentation, is central to colonization and persistence of this pathogen during infection. Here using activity-based protein profiling (ABPP), we revealed Stickland enzyme activity is a biomarker for *C. difficile* infection (CDI) and annotated two such cofactor-dependent Stickland reductases. We structurally characterized the cysteine-derived pyruvoyl cofactors of D-proline and glycine reductase in *C. difficile* cultures and showed through cofactor monitoring that their activity is regulated by their respective amino acid substrates. Proline reductase was consistently active in toxigenic *C. difficile*, confirming the enzyme to be a major metabolic driver of CDI. Further, activity-based hydrazine probes were shown to be active site-directed inhibitors of proline reductase. As such, this enzyme activity, *via* its druggable cofactor modality, is a promising therapeutic target that could allow for the repopulation of bacteria that compete with *C. difficile* for proline and therefore restore colonization resistance against *C. difficile* in the gut.

## 1 Introduction

*Clostridioides difficile*, previously known as *Clostridium difficile* (Lawson et al., 2016) (*C. difficile*), is a gram-positive, spore-forming, obligate anaerobic bacteria and a leading healthcare-associated disease (Lessa et al., 2015). *C. difficile* infection (CDI) and pathogenesis are driven by two toxins, toxin A (tcdA) and toxin B (tcdB), which cause a wide range of symptoms including diarrhea, pseudomembranous colitis, toxic megacolon and/or death (Rupnik et al., 2009; Carter et al., 2010). CDI is most commonly associated with antibiotic-induced perturbation to the resident gut microbiota and has high rates of recurrence (25%) (Belmares et al., 2009; Lessa et al., 2012; Lessa et al., 2015). Emergence of hypervirulent and antibiotic resistance strains (McDonald et al., 2005; Kelly and LaMont, 2008; Merrigan et al., 2010; He et al., 2013) and a rise in community (Ofori et al., 2018) and animal-associated (Knetsch et al., 2018) infections, highlight the public health challenge that this pathogen represents.

**FIGURE 1:**
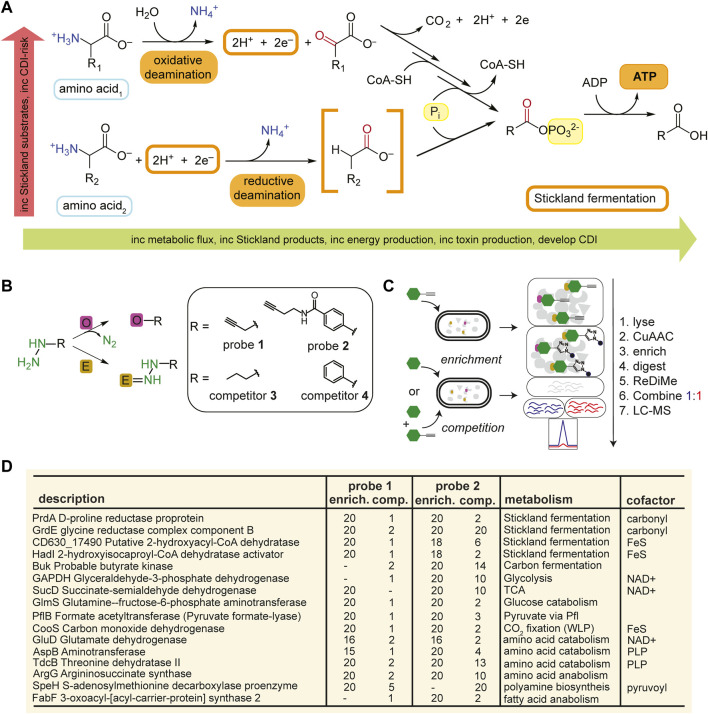
Identification of metabolic protein targets by hydrazine probes in *C. difficile*. **(A)**, Representative schematic of Stickland fermentation coupled oxidation and reduction of amino acids to generate ATP. **(B)**, Labelling mechanisms of electrophilic (E) and oxidative (O) cofactors by hydrazine. Hydrazine warheads are shown in green. R is structure of probe or competitor. See Supplementary Figure S3 for full structures. **(C)**, Schematic for MS^2^-based quantitative proteomics experiments (enrichment and competition) in living bacteria using late-stage reductive dimethylation. Isotopically heavy and light peptides are depicted in blue and red, respectively. **(D)**, High-reactivity protein targets of hydrazine probes in *C. difficile* associated with metabolic pathways shown in Supplementary Figure S1. See Supplementary Figure S6; Supplementary Datasets S1, S2 for complete protein target lists.

The ecological success of *C. difficile* is in part due to its unique, complex, and adaptable energy metabolism, which is tightly associated with virulence regulation (Karlsson et al., 2008; Neumann-Schaal et al., 2015; Hofmann et al., 2018). *C. difficile* utilizes sugars and amino acids as main sources of energy through many metabolic processes, (Neumann-Schaal et al., 2019), including central carbon metabolism and fermentation pathways (Supplementary Figure S1). Most of these processes are additionally utilized by other species, including the human host, or at least by other anaerobic bacteria; however, Stickland fermentation is an exception. Stickland fermentation is a *Clostridia*-specific amino acid fermentation where the oxidation and reduction of amino acids is coupled to produce ATP (Figure 1A) (Stickland, 1934). CDI-susceptible environments are characterized by an increase of amino acids (Battaglioli et al., 2018) with several studies correlating their abundance or their subsequent depletion through Stickland metabolism with CDI (Jenior et al., 2017; Robinson et al., 2019; Aguirre et al., 2021; Girinathan et al., 2021; Hofmann et al., 2021; Smith et al., 2022). Moreover, while Stickland reaction products are positively associated with CDI and toxin production, carbohydrate carbon sources for central carbon metabolism and fermentation are negatively correlated with CDI, suggesting a shift in the metabolome of *C. difficile* and a dependence on amino acid utilization through Stickland reactions during colonization and infection (Fletcher et al., 2018; Robinson et al., 2019).

Understanding *C. difficile* metabolism is important in understanding colonization of and colonization resistance against *C. difficile*. Recent literature shows that the *bai* encoding bacteria (e.g., *Clostridium scindens*, *Clostridium hiranonis*) that produce secondary bile acids, thought to be toxic to and therefore protective against CDI, also protect in a bile acid-independent manner through Stickland metabolism, particularly by glycine and proline utilization and 5-aminovalerate production, supporting competition for nutrients in addition to secondary bile acids as the mechanism of colonization resistance against *C. difficile* (Aguirre et al., 2021). Elucidating the roles of metabolism during colonization and infection of *C. difficile*, especially those of the Stickland reactions, provides promising therapeutic potential. Many metabolic enzymes utilize non-encoded, catalytically essential, post-translational modifications or cofactors. These acquired cofactors impose new functions onto individual proteins and in general expand the catalytic reach of the proteome (Davidson, 2018). In Supplementary Figure S2, representative electrophilic and oxidative cofactors utilized by metabolic enzymes of bacteria are presented including the post-translationally installed, protein-derived pyruvoyl (van Poelje and Snell, 1990), formylglycine (Appel and Bertozzi, 2015), and quinone (Klinman and Bonnot, 2014) cofactors, and incorporated pyridoxal 5’-phosphate (Liang et al., 2019) (active form of vitamin B_6_), riboflavin-derived flavin (flavin mononucleotide, or flavin adenine dinucleotide) (Sepulveda Cisternas et al., 2018) and iron containing, heme (Augusto et al., 1982) and iron-sulfur cluster (Ayala-Castro et al., 2008), cofactors. As these cofactors are regulated, they act as sensors for metabolic activity and can be tracked through chemoproteomic approaches like activity-based protein profiling (ABPP) (Cravatt et al., 2008) that capture them in their transient, but functional state. Recently we showed that hydrazine-based reverse polarity-ABPP (RP-ABPP) (Matthews et al., 2017; Dettling et al., 2020) probes are active site-directed inhibitors for many classes of enzyme targets dependent on electron deficient cofactors and can be developed into selective and potent inhibitors (Lin et al., 2021; Wang et al., 2022). Organohydrazines can covalently inactivate proteins with electrophilic cofactors *via* direct polar coupling (DPC) and proteins with electron deficient oxidative cofactors *via* radical oxidative fragment coupling (Figure 1B) (Lin et al., 2021). Hydrazine-based ABPP probes can be utilized to monitor and interrogate cofactor-dependent metabolic enzymes of *C. difficile* pathways and identify new therapeutic avenues for this genetically intractable infectious disease. Here we utilize hydrazine driven RP-ABPP to map the metabolic activity of *C. difficile* and to identify an enzyme drug target of Stickland fermentation both associated with infection and susceptible to chemical modulation.

## 2 Results

### 2.1 *In situ* profiling with hydrazine probes in *C. difficile*

As this is our initial application of RP-ABPP in *C. difficile* culture, we generated a global map of the hydrazine-reactive proteome with the original broadly targeting alkyl probe **1** and aryl probe **2** (Matthews et al., 2017) (Figure 1B; Supplementary Figure S3). **1**-and **2**-labelling conditions were optimized by gel-based profiling. Gel-based proteomic profiles of **1** and **2** were generated after anaerobic treatment of *C. difficile* VPI 10463 cells (cultivated in brain heart infusion, BHIS) in stationary phase (0.5 h, 37°C). After lysing, fractionation, and conjugation of probe-labelled proteins to rhodamine-azide (Rh-N_3_) reporter tags through copper (I)-catalyzed azide-alkyne cycloaddition (CuAAC or “click” chemistry) (Rostovtsev et al., 2002), the probe-captured proteins were visualized using SDS-PAGE with in-gel fluorescence scanning (Matthews et al., 2017). **1**- and **2**-labelling was suppressed by pretreatment (0.5 h, 37°C) with non-clickable competitors **3** and **4** (Supplementary Figure S4). In addition, *C. difficile* growth (optical density at 600 nm, OD_600_) and viability (colony forming unit, CFU) weren’t significantly affected by addition of hydrazine competitors **3** and **4** at treatment concentrations (Supplementary Figure S5). To identify and quantify the protein targets of **1** and **2**, *C. difficile* cells were treated with **1** (3 mM, 0.5 h, 3°C) or **2** (1 mM, 0.5 h, 37°C) anaerobically and then probe-captured proteins were conjugated to biotin-N_3_ by “click” chemistry, enriched by adsorption to streptavidin beads, digested with trypsin protease, and the resulting proteomes were ratiometrically compared after reductive dimethylation (ReDiMe) (Boersema et al., 2009) of tryptic peptides (Figure 1C). To assess enrichment, proteomes from probe-treated cells were compared to proteomes treated with competitor at the same concentration and to assess competition, proteomes from probe-treated cells were compared to proteomes pretreated with 10X competitor and then probe at the same concentration. Protein enrichment and competition ratios were determined by the median ReDiMe ratio of two or more unique quantified peptides and then averaged across replicates (*n* = 4 biological replicates). Average enrichment ratios were plotted against average competition ratios and those proteins that were both enriched by **1** or **2** (ratio ≥8) and in competition with **3** or **4** (ratio ≥2) were considered high-reactivity targets (Supplementary Figure S6; Supplementary Datasets S1, S2).

Many of the high-reactivity targets of **1** and **2** in *C. difficile* were metabolic enzymes distributed across the various metabolic processes utilized by *C. difficile* and possess known cofactors ranging from carbonyl pyruvoyl (Pvyl) cofactors to iron sulfur (FeS) clusters (Figure 1D). These targets validate that RP-ABPP can probe cofactor-dependent metabolic activity in a human enteric pathogen. Four enzymes part of the reductive branch of Stickland fermentation (Figure 1A) were high-reactivity targets, two of which are selenoenzymes, D-proline reductase (PR) and glycine reductase (GR), that reduce proline and glycine, respectively, through a modified Stickland pathway. PrdA and GrdE, the hydrazine reactive subunits of these protein complexes, encode for proenzymes that are post-translationally processed into two subunits each with one possessing an *N*-terminal carbonyl containing cofactor detected *in vitro* by fluorescein thiosemicarbazide (Kabisch et al., 1999; Andreesen, 2004). The presence of these carbonyl electrophiles in PR and GR are consistently noted as being essential for function (Kabisch et al., 1999; Wagner et al., 1999; Andreesen, 2004).

### 2.2 Exploring context-dependent activity of Stickland enzymes

**FIGURE 2:**
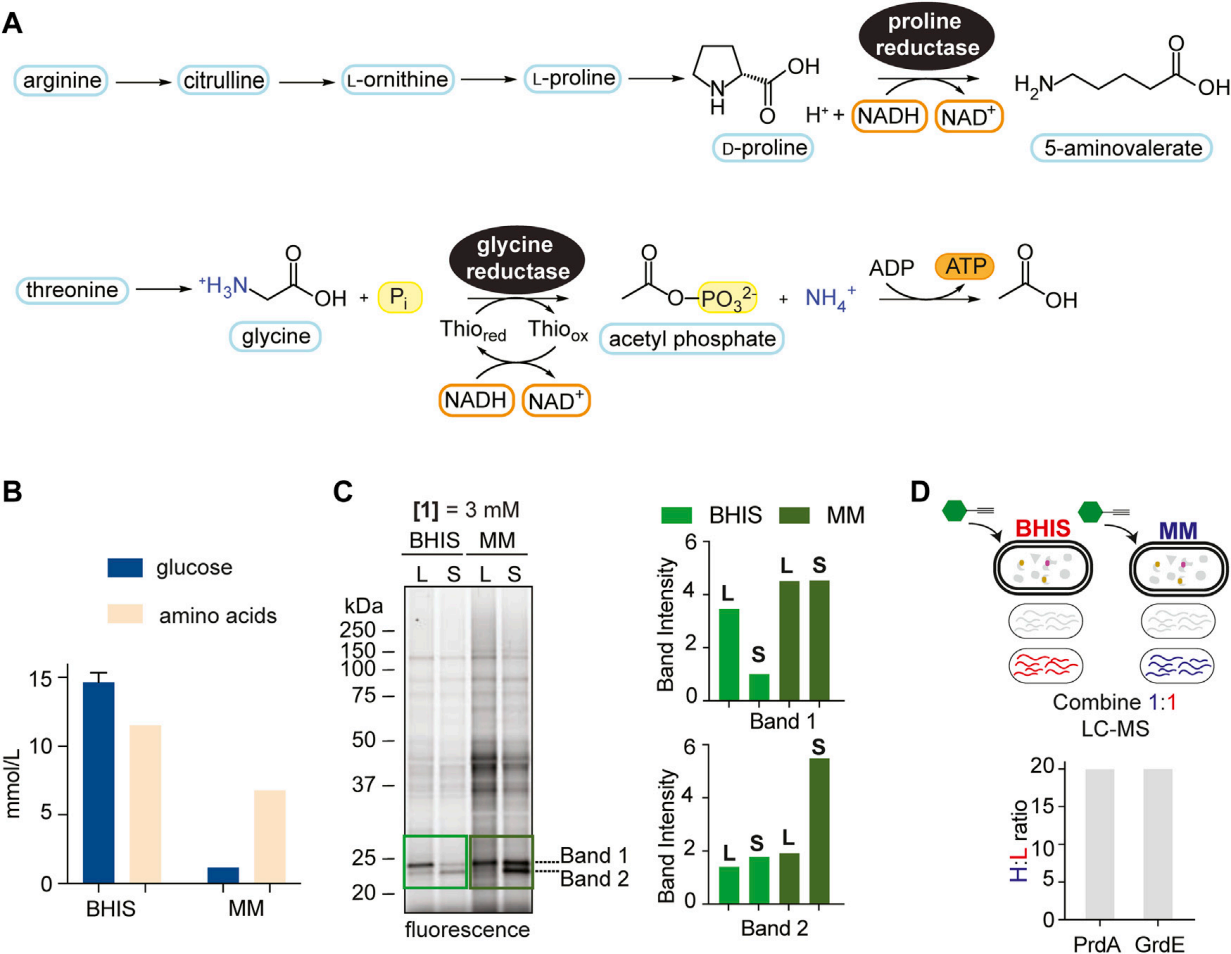
Media comparison showing context dependence of Stickland fermentation. **(A)**, The reduction of proline and glycine by PR and GR, respectively, through modified reductive Stickland pathways. **(B)**, Quantified amino acid and glucose concentrations (average of 3 technical replicates) (mmol/L) in BHIS and MM. See Supplementary Tables S2, S3 for more details. **(C)**, Gel-based labelling profiles of probe **1** in the soluble proteome of *C. difficile* cells cultivated in BHIS and MM harvested in mid-log phase (L) and stationary phase (S) (*left*) and relative band intensities of Band 1 and Band 2 in the 20–25 kDa region normalized to expression (*right*). Corresponding expression profiles are shown in Supplementary Figure S8. Intensity of Band 2 in BHIS is set to a relative intensity of 1. **(D)**, Comparison of PrdA and GrdE enrichment by hydrazine in BHIS and MM using late-stage reductive dimethylation. Ratios quantify and compare PrdA and GrdE activity in the different media environments. Averages from two technical replicates along with standard deviations are shown.

As Stickland fermentation is the dominant metabolism during colonization and initial stages of infection, we first applied RP-ABPP to explore PR and GR and their cofactor-dependent activities. PR catalyzes the reductive cleavage of the D-proline ring, which is derived from arginine (Fonknechten et al., 2010), to form 5-aminovalerate, which is excreted (Figure 2A) (Kabisch et al., 1999). PR is expected to have a role in redox balance as it was shown to be coupled to proton motive force (PMF) in *Clostridium sporogenes* potentially through the Rnf complex and therefore additionally involved in ATP production (Lovitt et al., 1986; Liu et al., 2022). GR is linked to PR through *prdR* (Bouillaut et al., 2013), which controls the proline interdependent regulation of both, and catalyzes the reduction of glycine to acetyl phosphate which directly transfers a phosphate group to ADP to produce ATP (Andreesen, 2004) (Figure 2A).

The onset of CDI is correlated to metabolic shifts of Stickland products (Fletcher et al., 2018; Robinson et al., 2019), a biochemical amplification associated with Stickland enzyme activity. RP-ABPP will serve as a tool to sense the cofactor-dependent activity of Stickland enzymes PR and GR and therefore allow real-time monitoring of Stickland fermentation metabolism upon which CDI is governed. We mimicked the shift to Stickland fermentation during colonization and infection, which is correlated to the shift away from central carbon metabolism (Fletcher et al., 2018; Robinson et al., 2019), in culture by utilizing growth medium that differs in their glucose and amino acid concentrations. A recent study only altering glucose availability showed the *C. difficile* intracellular metabolome shifts to amino acid metabolism in the absence of glucose (Hofmann et al., 2021), suggesting that the amino acid:glucose ratio is reflective of Stickland fermentation dependence. Here we utilized BHIS, a rich growth medium that supplies large quantities of sugar and amino acids, and a minimal medium (MM) (Seddon and Borriello, 1989) (Supplementary Table S1; Supplementary Figure S7), that limits sugar and is rich in amino acids, that have relative amino acid:glucose ratios of 5.8 and 0.8, respectively (Figure 2B; Supplementary Tables S2, S3), to characterize Stickland enzyme activity in the onset of disease. As MM is rich in amino acids, it is most physiologically relevant to the dysbiotic gut during colonization and infection (Battaglioli et al., 2018). *C. difficile* proteomes from cells cultivated in BHIS or MM and treated with **1** at mid-log phase or stationary phase were compared by gel-labelling. Overall, proteome-wide **1**-labelling was increased in MM, we expected because of divergent metabolic activities induced by nutrient availability (Figure 2C *left*). Expression profiles were consistent by Coomassie staining (Supplementary Figure S8) emphasizing ABPP’s ability to differentiate protein expression from activity. Strong activity, which increased in MM compared to BHIS in both log and stationary phase treatment, was conserved in two bands in the 20–25 kDa region, leading us to wonder if they correlated to the cofactor harboring PrdA (∼23 kDa) and GrdE (∼20 kDa) of PR and GR, respectively (Figure 2C *right*). In-gel digestion of the 20–25 kDa molecular weight region followed by ReDiMe (Boersema et al., 2009) labelling of the resulting tryptic peptides confirmed high-level expression (top 5% by spectral counting) of PrdA and GrdE that was comparable in both media conditions (Supplementary Figure S8; Supplementary Dataset S3). In studies looking at preferential amino acid utilization in the presence of glucose in *Clostridium sticklandii* (*C. sticklandii*) and *C. difficile*, proline was utilized immediately in log phase and glycine, though also utilized preferentially to other amino acids, was used slightly slower with ∼50% utilized by mid-log phase (Fonknechten et al., 2010; Neumann-Schaal et al., 2015). This could be reflective in Band 1 and Band 2 activity in BHIS in Figure 2C. In MM, however, the activity of these two bands don’t decrease as stationary phase is reached but stay constant or increase reflecting sustained/increased activity. In a parallel enrichment experiment where probe-treated proteomes of cells cultivated in MM were compared to probe-treated proteomes of cells cultivated in BHIS (stationary phase), a ∼20-fold increase in the fraction of hydrazine captured (active) PR and GR in MM compared to BHIS was observed (Figure 2D; Supplementary Dataset S4), supporting the sustained/increased amino acid utilization in sugar depleted environments seen by gel and moreover how Stickland fermentation sustains *C. difficile* during colonization and infection. Therefore, our data suggests that Stickland enzyme activity, such as PR and GR measurable here by RP-ABPP, are potential biomarkers for *C. difficile* colonization and infection and ultimately disease progression.

### 2.3 Structural characterization of carbonyl cofactors in PrdA and GrdE

As these Stickland reductases are cofactor-dependent, species-specific, and essential metabolic enzymes during disease, they have potential to be better pharmacological interventions in the age of antibiotic resistance. While vancomycin, fidaxomicin and metronidazole are standard antibiotic treatments for *C. difficile*, there is a high rate of infection reoccurrences and a high number of emerging hypervirulent strains resistant to standard care (He et al., 2013; Eubank et al., 2022; Olaitan et al., 2022). In addition, these antibiotics don’t address the cause of infection—the imbalance of the microbiome—but rather combat the effects. We sought to explore the small molecule reactivity of the carbonyl cofactors of PR and GR further, as capturing this chemistry in active cultures is necessary for therapeutic target evaluation and for the potential development of potent and selective active site-directed inhibitors.

**FIGURE 3:**
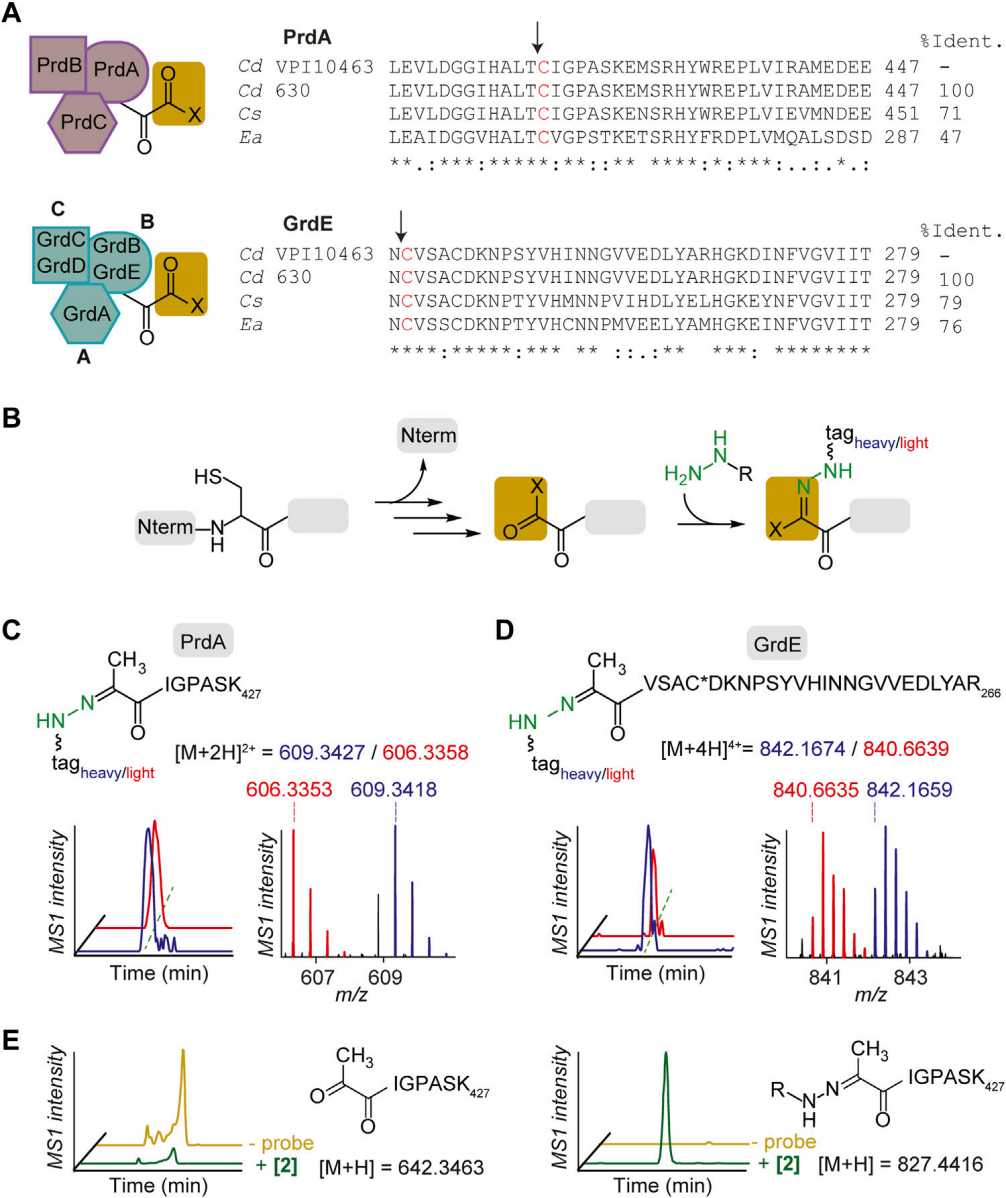
Characterization of hydrazine-reactive sites in PR and GR of Stickland fermentation. **(A)**, PR complex and GR complex protein subunits (*left*). Alignment of bacterial reductase subunits, PrdA and GrdE, with putative sites of proteolysis and formation of carbonyl groups (*right*). The arrows note sites of proteolysis. Sites of modification are shown in red. *Cd*, *Clostridioides difficile*; *Cs*, *Clostridium sticklandii*; *Ea*, *Eubacterium acidaminophilum*. **(B)**, Labelling mechanism of carbonyl cofactors by hydrazine through Schiff base formation. **(C)**, The identity of the probe **2**-labelled peptide (aa 421–427) in PrdA in culture. Extracted MS1 ion chromatograms and corresponding isotopic envelope of co-eluting heavy- and light-tagged peptides are shown in blue and red, respectively. **(D)**, The identity of the probe **2**-labelled peptide (aa 242–266) in GrdE in culture. C* is site of static modification by carbamidomethyl. Extracted MS1 ion chromatograms and corresponding isotopic envelope of co-eluting heavy- and light-tagged peptides are shown in blue and red, respectively. See Supplementary Figures S10–S17; Supplementary Table S4 for further characterization. **(E)**, Extracted parent ion chromatograms of the *N*-terminal Pvyl peptide of PrdA (*left*) and **2**-Pvyl peptide adduct (*right*) in the absence and presence of **2** *in vitro*. See Supplementary Figure S18 for further analysis.

PR and GR are multicomplex proteins (Figure 3A *left*). PR consists of the proprotein PrdA, which is processed to form the carbonyl-containing subunit, the selenocysteine (sec)-containing PrdB and electron transfer protein PrdC (Kabisch et al., 1999). GR consists of the sec-containing thioredoxin-reduced protein A (encoded by GrdA), substrate-specific protein B (encoded by sec-containing GrdB and proprotein GrdE that is processed to form the carbonyl-containing subunit) and acetyl phosphate-forming protein C (encoded by GrdC and GrdD) (Andreesen, 2004). PR and GR were first annotated as carbonyl-containing dependent enzymes in other Stickland fermenting organisms, *C. sticklandii* (Kabisch et al., 1999) and *Eubacterium acidaminophilum* (Wagner et al., 1999). Alignment of bacterial reductases PrdA and GrdE with putative sites of proteolysis and formation of carbonyl groups is shown in Figure 3A *right*. PrdA and GrdE possess cysteine-derived carbonyl groups based on previous detection with fluorescein thiosemicarbazide as mentioned and an absence of characteristic absorbances for other electron deficient cofactors such as flavin, pyridoxal phosphate or FeS centers (Kabisch et al., 1999; Wagner et al., 1999). Previous reports suggest the identity of the carbonyl group is a Pvyl cofactor (Kabisch et al., 1999; Bednarski et al., 2001) based on the well-known serine-derived Pvyl dependent enzymes that similarly encode for proenzymes that undergo autoproteolytic cleavage to generate two subunits, one of which possesses a Pvyl group at the *N*-terminus (van Poelje and Snell, 1990). PR and GR utilize their carbonyl cofactors to reduce their respective amino acid substrates through Schiff base formation (Kabisch et al., 1999; Wagner et al., 1999) similarly to the well characterized decarboxylases (van Poelje and Snell, 1990). Based on the Schiff base formation between both the decarboxylases and reductases with their substrates, and between hydrazine with carbonyl cofactors (formylglycine, glyoxylyl, Pvyl) (Appel and Bertozzi, 2015; Matthews et al., 2017), we expected hydrazine to covalently capture the carbonyl cofactors in PR and GR in similar Schiff base fashion (Figure 3B).

Utilizing isoTOP-ABPP (Weerapana et al., 2007; Matthews et al., 2017; Lin et al., 2021), a site-specific profiling method that utilizes isotopically differentiated protease cleavable biotin-azide tags that releases the probe-captured peptides as mass-differentiated pairs (Supplementary Figure S9), the *N*-terminal cysteine-derived cofactors of PrdA and GrdE were captured by probes in *C. difficile* culture and characterized by LC-MS/MS. The **2**-labelled peptides of PrdA (aa421-427) and GrdE (aa242-266) confirmed the presence of *N*-terminal cysteine-derived Pvyl cofactors through MS1 and MS2 spectra analysis (Figures 3C, D; Supplementary Figures S11, S13). Characterization was performed with **1** as well and in both BHIS and MM (Supplementary Figures S10–S17). Mass errors and statistics for reported MS1 pairs of all probe-labelled peptides in proteins studied here are summarized in Supplementary Table S4. This is the first structural characterization of PR and GR’s Pyvl activity captured in culture and one of the few known examples of a Pyvl cofactor derived from an internal cysteine, assigning PR and GR to their own subclass of Pvyl-dependent enzymes. *In vitro* capture of active PR purified by ammonium sulfate fractionation and hydrophobic interaction chromatography and yielding a final protein comparable to literature quality (Supplementary Figure S18) was performed as well. We observed loss of the unmodified *N*-terminal Pvyl tryptic peptide in **2**-treated PR protein (Figure 3E). No other tryptic peptides were modified in the **2**-treated protein supporting that hydrazine reacts specifically with the Pvyl group (Supplementary Figure S18). These data cement PR and GR Pyvl cofactors as druggable modalities that can be therapeutically modulated by covalent small molecules.

### 2.4 Monitoring regulation of reductase activity *via* cofactor status

**FIGURE 4:**
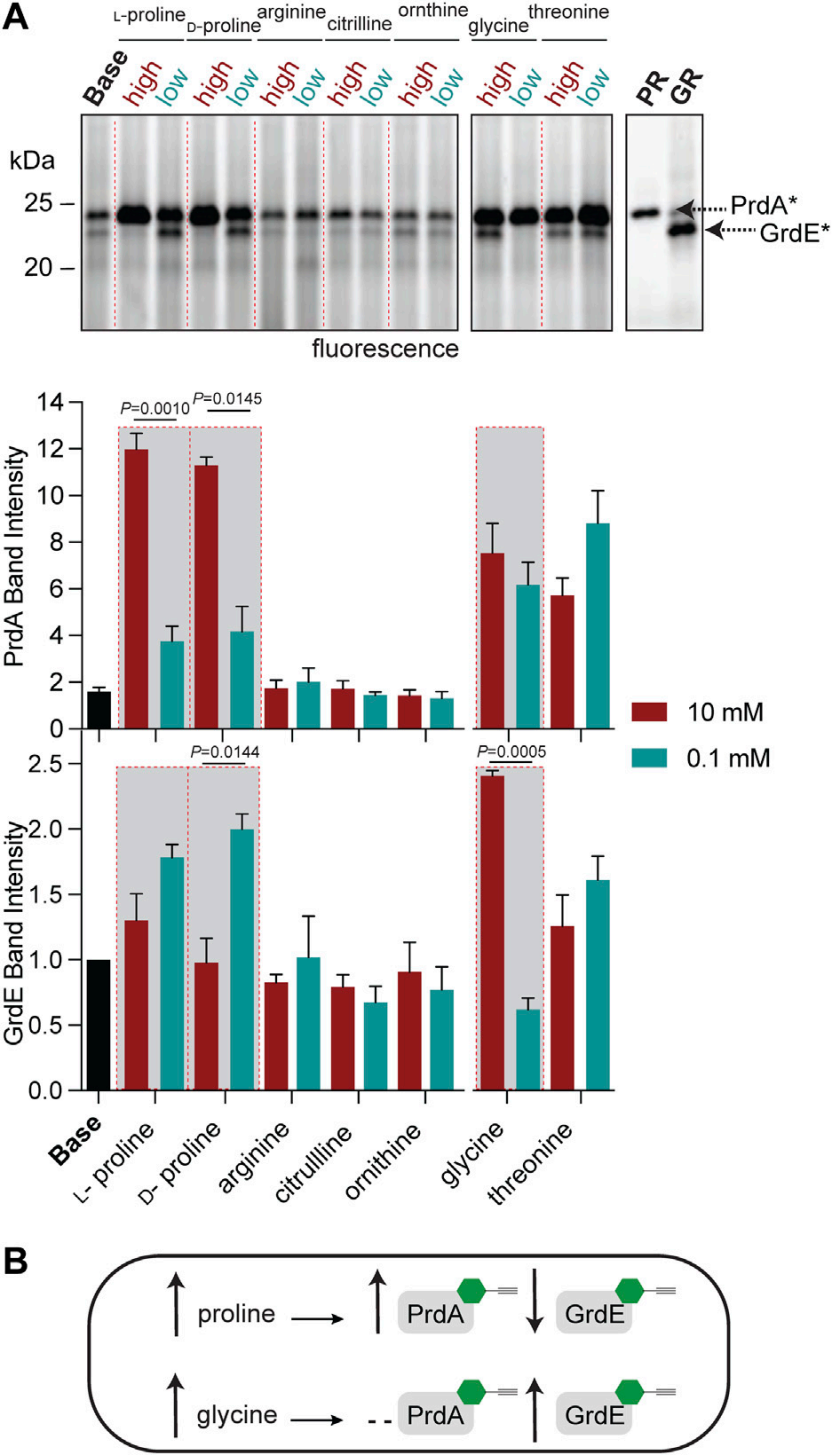
Functional profiling of the Pvyl cofactors of PrdA and GrdE by hydrazine probes. **(A)**, Gel-based monitoring of the fraction of Pvyl-PR and Pvyl-GR labelled by **2** in MM (Base) supplemented with high (10 mM) and low (0.1 mM) concentrations of selected metabolites (*upper*). **2**-Labelled purified PR and **2**-treated lysate of glycine-grown cells are included as controls. The normalized relative band intensities of Pvyl-PR and Pvyl-GR in the various media conditions normalized to expression, with Pvyl-GR in Base set to relative intensity of 1, are shown below (mean ± SEM, *n* = 3, *t*-tests with Welch’s correction using Prism9, *p* < 0.05 is significant). See Supplementary Figure S19 for full labelling gel and corresponding expression profiles. **(B)**, Schematic demonstrating the effects of proline and glycine on the fraction of Pvyl-PR and Pvyl-GR, emphasized in red-outlined boxes in **(A)**.

To further characterize PR and GR regulation beyond amino-acid rich environments, we applied RP-ABPP to map enzyme activity *via* cofactor status across multiple metabolite conditions. While the operon expression of *prd* and *grd* has been investigated (Bouillaut et al., 2013), cofactor-dependent activity in *C. difficile* cultures has not been elucidated to the best of our knowledge. In previous work, we utilized hydrazine to functionally profile *S*-adenosyl-ʟ-methionine decarboxylase (AMD1), a serine-derived Pvyl-dependent enzyme, by its Pvyl cofactor status, showing the Pvyl cofactor is metabolically regulated by its substrate precursor, ʟ-methionine (Matthews et al., 2017). To identify any specific amino acid substrate or precursors from the GR and/or PR pathway (Figure 2A) that directly regulates their Pvyl-dependent activity, we performed a media profile with MM (Supplementary Table S1) as the base. PR protein and glycine-grown *C. difficile* lysate acted as controls for gel-based investigation of PR and GR regulation*. C. difficile* cells were cultivated in low (0.1 mM) and high (10 mM) concentrations of selected metabolites, treated with **2** and the fraction of Pvyl-PR and Pvyl-GR captured by **2** was analyzed by gel (Figure 4A; Supplementary Figure S19). MM was supplemented with PR substrate precursors arginine, citrulline, ʟ-ornithine and ʟ-proline, PR substrate D-proline, GR substrate glycine and GR precursor threonine (Fonknechten et al., 2010) (Figure 2A). Pvyl-PR and Pvyl-GR band intensities were normalized to expression and Pvyl-GR was set to a relative intensity of 1. We found that high concentrations compared to low concentrations of ʟ-proline and D-proline increased the fraction of Pvyl-PR (*p* = 0.0010 and *p* = 0.0145, respectively), suggesting that proline upregulates both the expression and active fraction of PR protein (Figure 4A). D-proline pools are dependent on PrdF, part of *prd*, to convert ʟ-proline to substrate. No other metabolite tested affected PR activity selectively at high or low concentrations. Similarly, while investigating GR, we found that a high concentration of glycine compared to a low concentration increased the fraction of Pvyl-GR (*p* = 0.0005) suggesting that glycine upregulates both the expression and active fraction of GR protein. We were also able to observe the interdependent regulation between PR and GR as the active fraction of Pvyl-GR increased in low concentrations of D-proline compared to high (*p* = 0.0144), suggesting a reduction in the proline-dependent repression of *grd* (Bouillaut et al., 2013) (Figure 4A). These results, summarized in Figure 4B, suggest that PR and GR activity is consistent with gene operon; both are regulated by amino acid substrates but not pathway precursors. Additionally, this data shows that proline’s control of PR and GR persists, supporting that PR’s role is more important than GR and is a major metabolic driver of their respective fermentations and regulation.

### 2.5 Proline fermentation *via* Pvyl-PR is conserved

**FIGURE 5:**
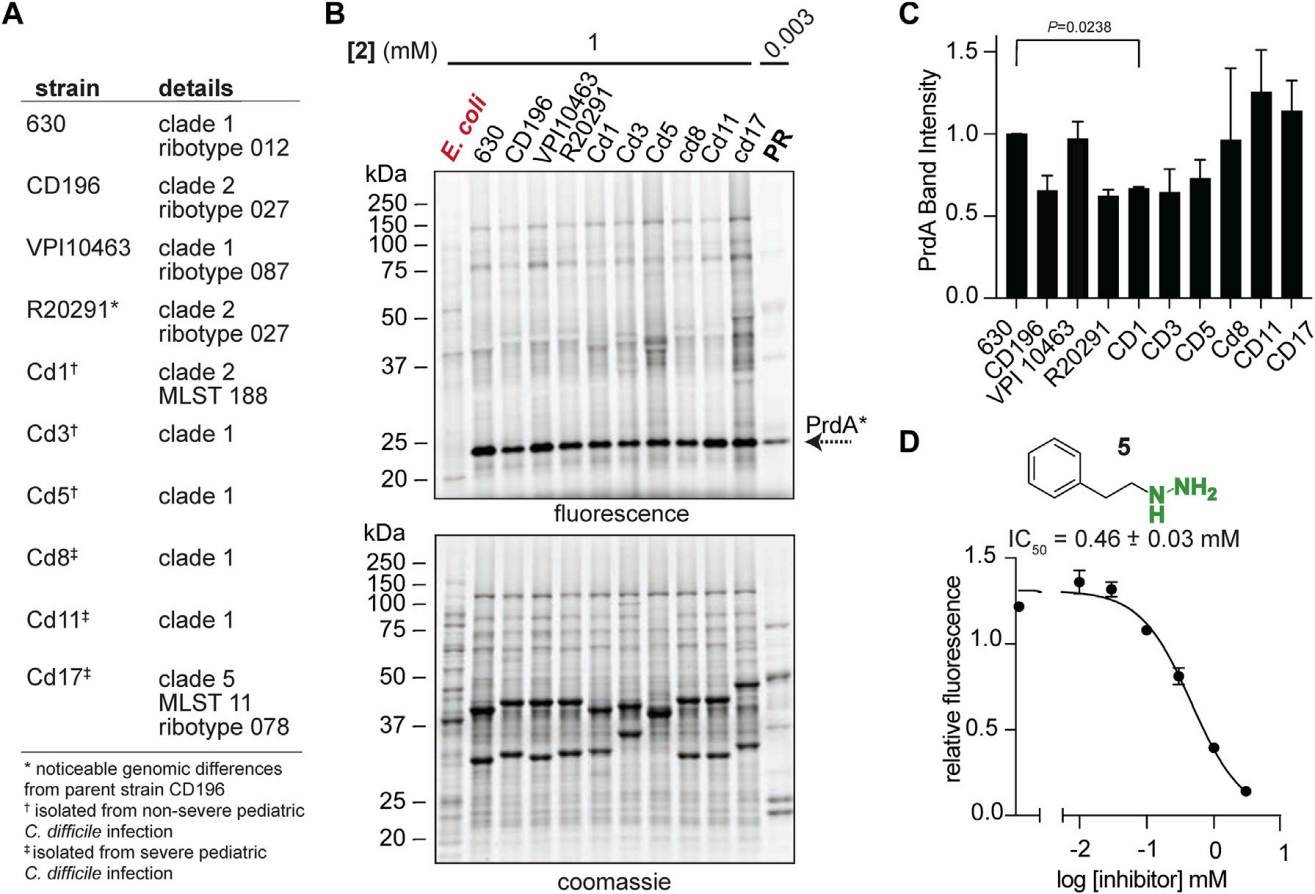
Pvyl-dependent PR activity is conserved. **(A)**, *C. difficile* laboratory strains, classical epidemic strains, and clinical strains isolated from pediatric patients with CDI. **(B)**, Gel-based proteomic profiles of *C. difficile* strains in **(A)** treated with **2** in mid-log phase (*upper*). **2**-labelled purified PR is included as a control. Corresponding expression profiles are shown after Coomassie staining (*lower*). **(C)**, Relative band intensities of Pvyl-PR in **(B)** normalized against expression. Pvyl-PR in strain 630 set to a relative intensity of 1 (mean ± SEM, *n* = 2, multiple pairwise *t*-tests using Prism9, *p* < 0.05 is significant). See Supplementary Figure S20 for stationary phase treatment. **(D)**, Inhibition curve of phenelzine (**5**) for PR. Averages from three independent experiments along with standard error are shown and fitted to a non-linear regression function [log (inhibitor) vs. response] in Prism 9. The IC_50_ value and standard deviation were calculated from non-linear regression functions from three independent experiments. See Supplementary Figure S21 for fluorescence assay schematic and additional inhibition curves.

To begin exploring PR as a new disease relevant enzyme drug target, we investigated PR activity in more physiologically relevant environments with the initial hope of observing a correlation between PR activity and CDI severity. Instead, we observed quite persistent PR activity across a broad sampling of laboratory strains, classical epidemic strains, and clinical strains isolated from pediatric patients with CDI (Bushman et al., 2020) (Figure 5A). Strains were cultivated in chemically defined medium (CDM) (Supplementary Table S5) and treated with **2** at mid-exponential phase and stationary phase. **2**-treated *E. coli* acted as a negative control as *E. coli* does not possess D-proline reductase as it doesn’t ferment amino acids through Stickland fermentation. At mid-exponential phase, Pvyl-PR was the most hydrazine reactive band across strains and the fraction of Pvyl-PR is roughly consistent, with no statistically significant differences between any of the severe and non-severe strains (Figures 5B, C). At stationary phase, Pvyl-PR lost some of is relative activity, but the fraction of Pvyl-PR present in all strains was still relatively consistent, with no statistically significant differences between any of the strains (Supplementary Figure S20). PR activity is expected to be dynamic and change throughout growth in response to proline availability and preference of utilization (Fonknechten et al., 2010; Neumann-Schaal et al., 2015), as reflected in Pvyl-PR’s reactivity compared to other enzymes in mid-log and stationary phase. It will be imperative to measure PR activity *in vivo* to account for host dependent nutrient availability. Regardless of these limitations, this data is powerful as it suggests PR is a consistently druggable target in toxigenic strains of *C. difficile*, independent of the strain or the severity of disease.

### 2.6 Cofactor targeting of proline reductase with organohydrazines

We have shown that our hydrazine-based chemical probes are activity-based tools to monitor and map PR activity. It is expected that organohydrazines, as they covalently modify the functional Pvyl cofactor, are active site-directed inhibitors of PR. PR activity was assayed by the Dithiothreitol (DTT) and PR-dependent production of 5-aminovalerate as previously described (Seto, 1979; Jackson et al., 2006). Detection of the fluorometric product of the reaction of 5-aminovalerate with *o*-phthalaldehyde (OPA), a reagent that forms a fluorogenic product with primary amines, was detected for reactions between 10 μg of purified PR with 10 mM D-proline with eight hydrazine-based inhibitors (*λ*_ex_ = 340 nm, *λ*_em_ = 455 nm) (Supplementary Figure S21). The relative fluorescence was proportional to the amount of enzyme present and in the absence of D-proline, no activity was observed (Supplementary Figure S21). We confirmed that organohydrazines from our established competitor library (Wang et al., 2022) such as **3**, **4**, phenelzine (**5**), an FDA-approved monoamine oxidase inhibitor (Zeller and Barsky, 1952), benzylhydrazine (**6**), O-phenylhydroxylamine (**7**), O-benzylhydroxylamine (**8**), 1-methyl-1-phenylhydrazine (**9**) and isoniazid (**10**), one of the first hydrazide-based drugs and current *Mycobacterium tuberculosis* treatment (Vilcheze and Jacobs, 2019), all inhibit PR’s utilization of D-proline as observed by a decrease in relative fluorescence (Figure 5D; Supplementary Figure S21). **7** and **8** form an oxime instead of hydrazone with Pvyl cofactors. Oxime formation is reportedly slower than hydrazone formation, but more stable as they are more resistant to protonation and subsequent hydrolysis (Kalia and Raines, 2008). Interestingly, **7** and **8** didn’t have a greater inhibitory effect than the hydrazone forming **4** and **6.** In general, the aryl (**4**, **9**) and alkylaryl (**5**) hydrazine compounds showed greater nucleophilicity and inhibition than the other compounds assayed, suggesting the conjugated π -systems contributed to the nucleophilicity of the warheads and stability of the resulting hydrazones. **5** had the greatest effect on substrate utilization (Figure 5D) with an IC_50_ 0.46 ± 0.03 mM. **4** and **6** performed slightly less well. Our data demonstrates that hydrazine probes not only serve as an *in vivo* measure for PR and GR activity, but they also are inhibitors that could be made to be more potent and selective, as previously shown (Wang et al., 2022).

## 3 Discussion

We have shown here that hydrazine ABPP probe technology is compatible with mapping metabolic enzymatic activity of pathogenic *C. difficile*. We have identified the hydrazine-reactive proteome in *C. difficile* and structurally characterized the hydrazine-reactive cofactors involved in the disease dominating Stickland fermentation metabolism, showing that PR and GR each contain catalytically essential cysteine-derived Pvyl cofactors that can be covalently inhibited by small molecules. This work not only shows the utility of RP-ABPP to identify and discover cofactors that are critical in a disease state, but also to track metabolism in the onset of infection through chemical capture of cofactor handles, revealing proline fermentation as a new therapeutic avenue to combat CDI.

We mapped Stickland reactions *via* PR and GR in the physiologically relevant MM observing amplification in Stickland enzyme activity that correlates to the metabolome shift during colonization and infection inferring Stickland enzyme activity is a sign of the initial stages of disease. Biochemical annotation of PR and GR showed that proline and glycine directly regulate the activity of PR and GR, respectively, uncovering a connection between expression and activity. However, we anticipate that there is more to uncover about their regulation, as we are uniquely interested in whether a fraction of PR and GR is inactivated during their catalytic cycle and if so, by what mechanism. Perhaps the Pvyl cofactor of theses Pvyl-dependent reductases are partitioned during catalysis to an inactive species, similar to the Pvyl cofactor of Pvyl-dependent decarboxylases, such as AMD1 (van Poelje and Snell, 1990). Further, previous work demonstrated that both GrdE and GrdB of GR interacted with fluorescein thiosemicarbazide, indicating two carbonyl-containing cofactors in GR (Wagner et al., 1999). The GrdB cofactor has a predicted role in glycine binding while the role of the Pvyl cofactor in GrdE is suggested to keep GrdB active (by transamination) if reduced (Andreesen, 2004). The location of GrdB’s predicted cofactor was not elucidated prior and in this work GrdB wasn’t a hydrazine-reactive target in *C. difficile* cultivated in BHIS. While efforts were made to characterize the predicted carbonyl cofactor in GrdB in *C. difficile* cultivated in BHIS and MM, we did not have success. If GrdB does possess an active site carbonyl cofactor or perhaps one of the other predicted functionalities (Andreesen, 2004), activity would be dependent on the regulation of two cofactors.

PR’s Pvyl cofactor is directly involved in proline binding as ^14^C proline has been found bound to the Pvyl-modified peptide (Kabisch et al., 1999), suggesting as shown here that hydrazine is an active site-directed inhibitor for proline fermentation through PR of toxigenic *C. difficile*. As genetic mutation of PR preventing proline fermentation through PR decreases both colonization and toxin production (Battaglioli et al., 2018), we suspect that pharmacological inhibition of PR will have a similar effect. We also observed persistent hydrazine-reactive PR activity in various toxigenic strains of *C. difficile*, suggesting Pvyl-PR is a consistent druggable modality for toxigenic *C. difficile*. It is possible that inherent differences in proline fermentation activity present in the host were then lost by cultivating them under the same media conditions outside the host. As such, investigating PR activity *in vivo* will be necessary to evaluate how host environment impacts PR’s persistence.

Proline is an essential nutrient for *C. difficile* (Jackson et al., 2006; Bouillaut et al., 2013; Theriot et al., 2014; Neumann-Schaal et al., 2015) and shows the largest increase in dysbiotic gut environments. (Battaglioli et al., 2018), while the proline fermentation product, 5-aminovalerate, has a positive correlation with CDI that increases significantly post infection (Fletcher et al., 2018; Lopez et al., 2019). This suggests that *C*. *difficile* is utilizing proline during infection, the source of which is likely diet, bystander microbiota, and toxin-induced host collagen degradation (Fletcher et al., 2021). Bacteria with similar nutritional requirements, such as non-toxigenic *C. difficile* and *bai* encoding *Clostridia*, have been shown to protect the host against toxigenic *C. difficile* (Gerding et al., 2015; Maslanka et al., 2020; Aguirre et al., 2021)*.* Though other *Clostridia* ferment proline through PR, data suggests the mechanism of PR in toxigenic *C. difficile* varies significantly (Jackson et al., 2006). We hypothesize that inhibition of PR of toxigenic *C. difficile* will impede *C. difficile*’s ability to compete with other microbiota for proline, enabling restoration of colonization resistance by facilitating the recovery of antagonistic microbiota (Aguirre et al., 2021) and ultimately facilitating clearance of infection. Our tools will allow for future monitoring of PR activity and inhibition in other proline-fermenting *Clostridia*, such as th*e bai* encoding *C. scindens,* to observe if the mechanism of PR in toxigenic *C. difficile* is different and if selective inhibition of PR in toxigenic *C. difficile* is both possible and necessary. Relatedly, future work will involve developing a potent and selective Pvyl-targeting compound for PR that can then be tested in CDI mouse models. As hydrazines can penetrate even the most isolated of spaces in the brain, they should be applicable for *in vivo* assessment of PR inhibition as a therapeutic avenue for CDI.

## 4 Materials and methods

### 4.1 Materials

All materials were obtained from commercial suppliers and used without further purification. Competitors **3**–**10** were purchased as follows: propylhydrazine dihydrochloride (**3**; Combi-Blocks), phenylhydrazine hydrochloride (**4**; Thermo Scientific), phenelzine sulfate salt (**5**; Sigma Aldrich), benzylhydrazine dihydrochloride (**6**; Combi-Blocks), O-phenylhydroxylamine (**7**; Thermo Scientific), O-benzylhydroxylamine (**8**; Combi-Blocks), 1-methyl-1-phenylhydrazine (**9**; TCI), isoniazid (**10**; Millipore Sigma). Alkylhydrazine probe (**1**) and phenylhydrazine probe (**2**) were synthesized according to literature (Matthews et al., 2017). Isotopic protease-cleavable biotin-azide peptide tags (TEV tags) for site of labelling experiments were synthesized as previously described (Matthews et al., 2017), adapted from previous procedure (Weerapana et al., 2007).

### 4.2 Stock solutions of hydrazine probes and competitors

Working stock solutions (0.1-0.3 M) of probe **1**, probe **2,** propylhydrazine **(3)** and phenylhydrazine **(4)** were prepared in H_2_O, with **2** and **4** containing 10% dimethyl sulfoxide (DMSO), according to previous procedure (Matthews et al., 2017). The stocks were neutralized to pH 6.0–7.0 and stored at—80°C prior to use.

### 4.3 Bacterial strains and growth conditions

*Clostridioides difficile* VPI 10463 (ATCC 43255) was sourced from Michael Abt (Department of Microbiology in the Perelman School of Medicine, University of Pennsylvania). Routine culture of *C. difficile* (Edwards et al., 2013) was carried out in brain heart infusion broth (BD Life Sciences) supplemented with 5 mg/mL yeast extract (Acros Organics) and 0.1% ʟ-cysteine (Alfa Aesar) (BHIS) until stationary phase (optical density at 600 nm, OD_600_, of ∼1.5) or mid-log phase (OD_600_, of ∼0.75). OD_600_ was measured using a spectrophotometer unless otherwise noted. *C. difficile* BHIS cultures were subcultured (1:100) into Minimal Medium (MM) and grown to stationary phase (OD_600_, of ∼0.5–0.6) or mid-log phase (OD_600_, of ∼0.2–0.3), where appropriate. MM was prepared according to a previously described recipe (Supplementary Table S1) (Seddon and Borriello, 1989). In brief, appropriate amounts were dissolved in distilled H_2_O (dH_2_O) and filter sterilized (0.2 µm pore size). Glucose concentrations in BHIS and MM were detected using a glucose meter (Gerrmaine™ Laboratories AimStrip ™ Plus Blood Glucose Testing System). Control solutions of 15 mM and 5 mM glucose were also recorded to assess if the meter and strips were working properly (Supplementary Table S2). Amino acids were quantified in BHIS and MM by the Microbial Culture and Metabolomics Core of PennCHOP Microbiome Program (Supplementary Table S3). Samples were derivatized using the Waters AccQ-Tag Ultra Amino Acid Derivatization Kit (Waters Corporation) and analyzed using the UPLC AAA H-Class Application Kit (Waters Corporation) on a Waters Acquity uPLC System with an AccQ-Tag Ultra C18 1.7 μm 2.1 × 100 mm column and a Photodiode Detector Array. Rich growth medium containing 20 g/L tryptone (dot scientific), 10 g/L yeast extract, 1.75 g/L K_2_HPO_4_ (Fisher), 1 µM selenite (Honeywell) and supplemented with 40 mM ʟ-proline (Alfa Aesar) and 40 mM ʟ-alanine (Alfa Aesar) or 50 mM glycine (Thermo Fisher Scientific) and 25 mM alanine was used to enhance expression of *prd* ad *grd.* (Meyer et al., 1995; Jackson et al., 2006). For solid medium, brain heart infusion agar was supplemented with 5 g/L yeast extract, 1% ʟ-cysteine, 250 mg/L D-cycloserine (MilliporeSigma), 8 mg/L cefoxitin (MilliporeSigma), and 1% taurocholic acid (Sigma) (CCBHIS-TA) (Sorg and Dineen, 2009). All *C. difficile* cultures were incubated in an anaerobic chamber (Type B, Coy Labs) at 37°C supplied with gas from a 85:5:10 N_2_:H_2_:CO_2_ Gas mix. All media and reagents were degassed in the anaerobic chamber for at least 24 h before use.

### 4.4 Treatment of *C. difficile* cells with hydrazine probes

Probe treatment was adapted and optimized following a previous study (Matthews et al., 2017). Briefly, *C. difficile* VPI 10463 cultures were grown anaerobically as described, harvested (5,000 g, 5 min, 4°C) and resuspended in 1 mL of growth media (BHIS, MM or CDM). The cells were treated with the probe (3 mM and 1 mM for **1** and **2**, respectively) and incubated anaerobically for 30 min at 37°C. For competition labelling experiments, cells were pretreated with non-clickable competitor (30 mM and 10 mM for **3** and **4**, respectively) for 30 min at 37°C and then treated with probe. After treatment the cells were washed by centrifugation (17,000 g, 3 min, 4°C) and resuspended in media (1 mL). Cell pellets were stored at—80°C prior to use.

### 4.5 Effects of probe treatment on *C. difficile* cell growth and viability

Continuous growth curves were performed in a microaerobic chamber (Coy) of the Microbial Culture and Metabolomics Core part of the University of Pennsylvania’s Children’s Hospital of Philadelphia (UpennCHOP) Microbiome Program. 4 µL of VPI 10463 BHIS starter culture was used to inoculate 200 µL of BHIS or MM in a 96-well plate. **3** and **4** were added in 3-fold dilutions from 3.0 to 0.01 mM to observe any effect on *C. difficile* growth. The OD_600_ was recorded using an Epoch2 plate reader (Biotek) every 15 min for 24 h, at 37°C with constant orbital shaking. Cell viability was quantified based on colony forming units (CFU) in the presence or absence of **3** and **4**. 10-fold serial dilutions of *C. difficile* BHIS or MM cultures, treated with **3** and **4** as described previously or treated with NaCl or 20% ethanol (Edwards et al., 2016), were plated anaerobically at 37°C on CCBHIS-TA agar. CFU was enumerated 24 h later. CFU/mL were calculated and averaged across replicates.

### 4.6 Proteome preparation of cells for gel- and MS-based experiments

Pelleted *C. difficile* cells were resuspended in 8 M urea in PBS (400 μL), incubated on ice for 30 min and lysed with a Branson SFX250 Sonifier equipped with a 102C (3 × 10 pulses, 0.3 s on and 2 s off, 15% energy). Soluble proteomes were fractionated by centrifugation (100,000 g, 30 min, 4°C) and total protein concentrations were determined by DC protein assay (Bio-Rad) on a microplate reader (Biotek ELx808).

### 4.7 Gel-based analysis of probe-labelled proteins

Gel-based analysis of probe-labelled proteins was performed as previously described (Matthews et al., 2017; Lin et al., 2021). A freshly prepared copper(I)-catalyzed azide-alkyne cycloaddition (CuAAC or “click”) reagent mixture (6 µL) containing 3 μL of 1.7 mM Tris (benzyltriazolylmethyl) amine (TBTA) in DMSO:*t*-BuOH (1:4 v/v), 1 μL of 50 mM CuSO_4_ in H_2_O, 1 μL of 1.25 mM rhodamine-azide (Rh-N_3_) in DMSO, and 1 μL of freshly prepared 50 mM tris(2-carboxyethyl) phosphine (TCEP) in H_2_O was added to samples (50 µL) containing the probe-labelled proteomes (1 mg/mL). After addition of the click mixture, samples were vortexed and incubated at room temperature for 1 h while rotating and quenched by addition of 17 μL of 4X sodium dodecyl sulfate (SDS) loading buffer. Probe-labelled proteins were resolved by SDS-PAGE and visualized by in-gel fluorescence scanning on a ChemiDoc MP Imaging System (Bio-Rad).

### 4.8 Sample preparation of ReDiMe experiments

A freshly prepared “click” reagent mixture (55 μL) was added to samples (500 μL) containing probe-labelled proteomes (1 mg/mL) for final concentrations of 100 μM TBTA, 1 mM CuSO_4_, 100 μM biotin-azide (10 mM in DMSO), and 1 mM TCEP. After addition of the click mixture, samples were vortexed, incubated at room temperature for 1 h while rotating, transferred to snap-closed falcon tubes on ice and quenched with the addition of pre-chilled methanol (MeOH, 1 mL), chloroform (CHCl_3_, 0.25 mL), and PBS (0.5 mL). The precipitated proteomes were centrifuged (5,000 g, 10 min, 4°C) to create protein disks, that were washed with 1:1 CHCl_3_:MeOH (3 × 1 mL). The protein disks were solubilized by mild sonication in cold 4:1 MeOH:CHCl_3_ (1.25 mL) and proteins were pelleted (5,000 g, 10 min, 4°C).

Protein pellets were solubilized by mild sonication in a freshly prepared solution of proteomics-grade urea (500 μL, 6 M in PBS). Disulfides were reduced with TCEP (final concentration: 9 mM) pre-neutralized with potassium carbonate (final concentration: 27 mM) for 30 min at 37°C. Reduced thiols were then alkylated by iodoacetamide (final concentration 45 mM) for 30 min at room temperature protected from light. SDS [10% w/v] was added to each sample to ensure complete denaturation and then the samples were diluted to a final concentration of 0.2% SDS in PBS. Samples were incubated with pre-equilibrated streptavidin beads (25 μL per sample, 100 μL 1:1 slurry, Pierce) to enrich probe-labelled proteins. After incubation, the beads were precipitated by centrifugation (1,400 g, 2 min) and washed with 0.2% SDS in PBS (3 × 5 mL), PBS (3 x 5 mL), and H_2_O (3 × 5 mL) to remove all impurities including unenriched proteins and detergents. The beads were transferred to new LowBind tubes (Eppendorf) and the enriched proteins were digested on bead overnight (8–12 h) at 37°C in 200 μL of 2 M urea containing 2 μg sequencing grade trypsin (Promega), 100 mM triethylammonium bicarbonate (TEAB) diluted in H_2_O, and 1 mM CaCl_2_.

After trypsin digestion, late-stage reductive dimethylation (Boersema et al., 2009) (ReDiMe) was performed. To the digested peptides of probe-treated cells, enriched “heavy” formaldehyde (^13^CD_2_O, Sigma Aldrich; 0.15%) was added and to digested peptides of competitor-treated (enrichment) or competitor- and probe-treated (competition) cells, naturally abundance formaldehyde (CH_2_O) was added at the same concentration. The N-termini and lysine residues of digested peptides were sufficiently dimethylated in the presence of sodium cyanoborohydride (NaBH_3_CN; 22 mM) after incubation at room temperature for 1 h. The reactions were quenched with ammonium hydroxide (0.13%) and the remaining trypsin was inactivated with formic acid (5%). The heavy- and light-derivatized samples were then combined and peptides were transferred to new tubes and stored at—80°C or desalted (see below) immediately.

### 4.9 isoTOP-ABPP sample preparation to isolate probe-captured peptides

To identify the probe-labelled peptides, the previously described isoTOP-ABPP protocol (Lin et al., 2021) adapted from previous studies (Weerapana et al., 2007; Matthews et al., 2017) was performed. Probe-labelled proteomes were diluted to 1 mL of 2 mg/mL in PBS and half of the proteome (0.5 mL) was conjugated to the light TEV tag and the other half to the heavy TEV tag. A freshly prepared “click” reagent mixture (60 μL) was added to each sample for final concentrations of 100 μM TBTA, 1 mM CuSO4, 100 μM light or heavy biotin-TEV-azide (5 mM in DMSO), and 1 mM TCEP. Mixtures were vortexed and incubated at room temperature for 1 h. Heavy and light-tagged proteomes were combined and proteins were pelleted (17,000 g, 2 min, 4°C) and then solubilized by mild sonication and washed by centrifugation in ice-cold methanol (2 × 0.5 mL). Proteins were solubilized by 1.2% SDS (1 mL in PBS) by sonication, diluted to 0.2% SDS with PBS (∼6 mL) and incubated with pre-equilibrated streptavidin agarose resin (50 μL per sample, 100 μL 1:1 slurry) for 3 h on rotator at room temperature. The resin was washed as described above in ReDiMe experiments, transferred to new LowBind tubes (2 × 500 μL H_2_O) and resuspended in 6 M urea (500 μL in PBS). Cysteines were reduced and alkylated as described above. The resin was washed with PBS (2 × 1 mL) to remove excess reagents and the enriched proteins were digested with trypsin (2 μg) overnight (8–12 h) at 37°C in the presence of 2 M urea (200 μL, in PBS) and CaCl_2_ (1 mM).

Unmodified peptides, urea, and trypsin were removed by sequential washes with PBS (9 × 0.5 mL). The resin was transferred to clean microcentrifuge tubes and equilibrated with TEV buffer (50 mM Tris, pH 8). Remaining immobilized peptides were released with TEV protease (∼2.2 µM in ∼335 µL TEV buffer at 30°C for 6 h). TEV proteolytic peptides containing heavy- and light-TEV tags were transferred to new tubes and recovered from the resin with H_2_O (2 × 50 µL). Sample was desalted immediately (see below) without addition of formic acid or stored at—80°C prior to desalting.

### 4.10 Sample analysis by liquid chromatography-tandem mass spectrometry (LC-MS/MS)

Peptide samples were desalted prior to LC-MS/MS analysis by using in-house packed stage-tips and then analyzed on in-house C18 packed nano-columns according to previous procedure (Lin et al., 2021) using methods adapted from previous studies (Weerapana et al., 2007; Weerapana et al., 2010; Matthews et al., 2017; Lin et al., 2021). In brief, peptides were analyzed using an LC-MS/MS system of an Easy-nLC 1,200 coupled to a Fusion Orbitrap (Thermo Scientific). Peptides from ReDiMe experiments were separated by a mobile phase linear gradient of A (0.1%FA in H_2_O) and B (80% acetonitrile in H_2_O containing 0.1% FA) under the following conditions: 0  →  5  →  55  →  65  →  85 min, 2% →  8% →  35% →  100% →  100% B. Peptides from IsoTOP-ABPP experiments were separated and eluted under the following conditions: 0  →  5  →  60  →  70  →  100 min, 0% →  0% →  45% →  100% →  100% B. 5 µL of each ReDiMe and isoTOP-ABPP sample was injected. Peptides from pure protein samples were separated and eluted under the following conditions: 0  →  10  →  12  →  21 min, 5% →  40% →  100% →  100% B. All samples were eluted with a flow rate of 300 nL/min, except for GrdE peptides from IsoTOP-ABPP which were eluted with a flow rate of 500 nL/min. All other parameters were consistent with previous procedure (Lin et al., 2021).

### 4.11 Peptide and protein identification and quantification

Each data file (in “.raw” format) was generated by the instrument (Xcalibur software). A derived file (in “.ms2” format) containing MS2 spectra for all fragmented parent ions was extracted using RawConverter (version 1.1.0.23) with monoisotopic selection (2015 released, publicly available at http://fields.scripps.edu/rawconv). Each “.ms2” file was searched using the ProLuCID algorithm against a reverse-concatenated, non-redundant database of the *C. difficile* VPI 10463 proteome (NCBI release −05/13/21) and filtered using DTASelect 2.0 within the Integrated Proteomics Pipeline (IP2) software. Cysteine residues were searched with a static modification for S-carbamidomethylation (+57.02146 Da). Methionine residues were searched with up to one differential modification of oxidation (+15.9949 Da). Peptides were required to have at least one tryptic terminus but an unlimited number of missed cleavages. For ReDiMe samples, the searches allowed for dimethylation of lysine residues (+28.0313 K) and the N-terminus (+28.0313 N-term), and isotopic heavy dimethylation of lysine and the N-terminus (+6.03181 K, +6.03181 N-term). The parent ion mass tolerance for a minimum envelope of three isotopic peaks was set to 50 ppm, the minimum peptide length was six residues, the false-positive rate was set at 1% or lower, and a minimum of two peptides per protein was required to be advanced to the next step of analysis. Heavy and light parent ion chromatograms associated with successfully identified peptides were extracted and compared using in-house software (CIMAGE) as previously described (Weerapana et al., 2010) (script is publicly available to download from GitHub at https://github.com/radusuciu/cimage-simple). Peptides and proteins were quantified using CIMAGE and high-reactivity targets were identified using postCIMAGE script, publicly available to download from GitHub at https://github.com/BeckyHan/Matthews-Lab/blob/master/postCIMAGE.R, as previously described (Lin et al., 2021), except that protein enrichment and competition ratios were determined by the median ratio from two or more unique quantified peptides, instead of three or more. Protein enrichment and competition ratios were then averaged across four biological replicates to generate the final average enrichment and competition ratios for each protein. The final data are reported in Supplementary Datasets S1, S2.

For the identification of probe-labelled proteins by isoTOP-ABPP, data was extracted and searched as described above. An additional Java script, MS2SpecFinder, was utilized to search the plausible MS2 spectra of a given peptide from the MS2 generated file. The code is publicly available to download from GitHub at https://github.com/matthewslab/probe. To calculate the statistics of monoisotopic masses of probe-labelled peptides in MS1 identified using Iso-TOP-ABPP (Supplementary Table S4), a custom *R* script, MS1_find, publicly available to download from GitHub at https://github.com/BeckyHan/Matthews-Lab/blob/master/MS1_find.R, was utilized to extract the experimental MS1 values using the theoretical monoisotopic values.

### 4.12 Quantification of PrdA and GrdE expression and activity in BHIS and MM

For the quantification of PrdA and GrdE expression, *C. difficile* soluble proteomes were diluted to 2 mg/mL and analyzed by gel as described above. The gel was stained with Coomassie dye that is MS-compatible (GelCode Blue Stain) and rinsed thoroughly. The 20–25 kDa expression region was manually excised and digested following previous procedure (Matthews et al., 2017). Briefly, gel pieces were washed with 100 mM ammonium bicarbonate (2 × 0.5 mL) and dehydrated with acetonitrile until the gel pieces were completely opaque. Cysteines were alkylated by rehydration with iodoacetamide (55 mM in 100 mM ammonium bicarbonate) for 30 min at room temperature protected from light. Gel bands were dehydrated again with acetonitrile and the gel-bound proteins were digested by rehydration with 0.4 μg trypsin (reconstituted in PBS) and further diluted to ∼200 μL with 25 mM TEAB overnight at 37°C). Late-stage reductive dimethylation (Boersema et al., 2009) with formaldehyde isotopologues as described above was used to differentiate growth conditions. Here, “heavy” formaldehyde was added to proteolyzed proteins isolated from MM growth conditions whereas “light” formaldehyde was added to proteolyzed proteins isolated from BHIS conditions. The samples were analyzed by LC-MS/MS as described above. For the quantification of PrdA and GrdE activity, soluble proteomes from treated cells were diluted to 1 mg/mL PBS and prepared as described above for ReDiMe experiments. Again, here “heavy” formaldehyde was added to proteolyzed treated proteins isolated from MM growth conditions whereas “light” formaldehyde was added to that from BHIS conditions. The samples were analyzed by LC-MS/MS as described above.

### 4.13 Purification of D-proline reductase from *C. difficile*

The purification of D-proline reductase was adapted from previous study (Jackson et al., 2006). *C. difficile* VPI 10463 was cultivated in rich growth medium to enhance the expression of *prd* at 37°C. After 24 h (OD_600_ = 1.4), cells were harvested by centrifugation (5,000 g, 30 min, 4°C), flash-frozen in liquid N_2_ and stored at—80°C. The cell paste (8–10 g/L culture) was resuspended (5 mL/g paste) in 50 mM Tris buffer (pH 8.4) containing 1 mM Ethylenediaminetetraacetic acid (EDTA), and 1 mM dithiothreitol (DTT) at 4°C, lysed by sonication (1 s on and 2 s off for 10 min, 40% amplitude) on Qsonica Q700 Sonicator and centrifuged (30,000 g, 20 min, 4°C). Stepwise ammonium sulfate fractionation (25%, 40%, 60%, and 85% saturation) was performed to isolate D-proline reductase-containing fractions. In brief, solid ammonium sulfate (Alfa Aesar) was slowly added to lysate stirring at 4°C to create a 25% saturated solution. After 1 h, precipitated proteins were collected by centrifugation (30,000 g, 20 min, 4°C). The above steps were repeated on the remaining supernatant to fractionate precipitated proteins at 40%, 60%, and 85% ammonium sulfate saturation. D-proline reductase was precipitated in the 40%–60% ammonium sulfate. This precipitate was solubilized in 50 mM Tris buffer (pH 8.4) containing 1 mM EDTA, 1 mM DTT, and 2 M ammonium sulfate and loaded onto a phenyl-Sepharose column (20 by 2.5 cm) equilibrated in the same buffer. The column was washed with equilibration buffer until absorption of the eluate at 280 nm (A_280_) and 260 nm (A_260_) was ∼0 (∼5 column volumes). Bound proteins were eluted by a 400 mL linear gradient of decreasing ammonium sulfate (2.0 M–0.0 M) in 50 mM Tris buffer (pH 8.4) containing 1 mM EDTA and 1 mM DTT. Fractions containing D-proline reductase as determined by SDS-PAGE were pooled and dialyzed against 50 mM Tris buffer (pH 8.4) containing 1 mM DTT and 250 mM NaCl for 12 h at 4°C. The protein was concentrated to ∼4 mg/mL in 10% glycerol and flash-frozen—80°C. The yield for D-proline reductase complex was 3 mg of protein per gram of cell paste. The active protein was confirmed by LC-MS/MS.

### 4.14 Probe 2-labelling of the pyruvoyl cofactor in PrdA *in vitro*

A 100 μL solution of 160 μg purified D-proline reductase in PBS was incubated overnight at room temperature in the presence (1 mM) and absence of probe **2** according to a previous procedure (Matthews et al., 2017). Protein in a 25 μL aliquot of the reaction was precipitated in 4:4:1 MeOH:H_2_O:CHCl_3_ (675 μL total) by centrifugation (17,000 g, 5 min, 4°C) into a protein disc, and was washed with MeOH (2 × 300 μL), solubilized by mild sonication and pelleted. Pelleted protein was solubilized in 50 μL PBS by mild sonication and half of the material (20 μg) was diluted with freshly made 6 M urea in PBS. Disulfides were reduced with TCEP (5 mM) pre-neutralized with potassium carbonate (15 mM) for 30 min at 37°C. Reduced thiols were alkylated with iodoacetamide (10 mM) for 30 min at room temperature protected from light. The solution was diluted to 2 M urea with PBS and digested for 3 h with 2 μg of trypsin in the presence of CaCl_2_ (1 mM). The remaining trypsin was inactivated by Formic acid (final concentration 5%). 3 μg of sample was desalted and 50 ng (2 μL) was injected into LC-MS/MS system. Note that for these experiments, peptides in the +1 charge state weren’t excluded for fragmentation.

### 4.15 Characterization of the PrdA *N*-terminus and reaction with probe

PrdA peptides were identified as described above except that the heavy search was excluded. The pyruvoyl *N*-terminal peptide before and after probe labelling was identified using differential modifications of −90.02517 (pyruvoyl), and 95.07013 (probe **2**-pyruvoyl) Da on cysteine with static *S*-carbamidomethylation (+57.02146 Da). In addition, these spectra were confirmed manually by extracting parent ion chromatograms (*m/z*_theor_ = 642.3457 and 827.4416, respectively, ±10 ppm) from the raw file, validating the corresponding isotopic envelopes reflect a peptide in the +1 charge state and assigning the fragment ions in the corresponding MS2 spectra (data not included). Parent ion chromatograms from unmodified peptides identified in the standard search were extracted from the raw file as well to compare the relative peak intensities of internal PrdA peptides in absence or presence of **2**. Individual IP2 searches for **2**-modified amino acids were also performed using a differential modification of +201.09021 (probe **2** labelled). Importantly, no other probe modified peptides were identified from this search. In addition, our, MS2 SpecFinder script (described above), was utilized to identify MS2 spectra for every peptide that showed variable differences in peptide intensity in the absence and presence of probe. Only MS2 spectra associated with unmodified forms of the peptides were found. These data showed that parent ion intensity differences observed between the tryptic peptides in the absence or presence of **2** aren’t a result of probe reactivity. In most cases, these differences could be attributed to variable miscleavages of trypsin.

### 4.16 Dynamic regulation of cofactor status in PrdA and GrdE

*C. difficile* VPI 10463 cells were cultivated in MM supplemented with high (10 mM) and low (100 μM) concentrations of potential regulatory metabolites (arginine, Acros Organics; citrulline, Acros Organics; ornithine hydrochloride, Alfa Aesar; ʟ-proline, D-proline, Acros Organics; threonine, Acros Organics; and glycine). Probe **2**-labelling and proteome preparation was performed as described above. Purified D-proline reductase and *C. difficile* VPI 10463 cells cultivated in rich growth medium to enhance the expression of *grd* (Meyer et al., 1995) were used for PrdA and GrdE band identification. D-proline reductase (0.3 mg/mL), as purified above, was incubated with probe **2** (3 μM, 15 min, 37°C) while glycine-grown *C. difficile* cells were treated with **2** as described above. All samples were subjected to fluorophore conjugation and gel-based analysis as described above. Band intensities of the active (probe-labelled) PrdA and GrdE subunits were quantified using ImageJ software (Schneider et al., 2012) and normalized against corresponding expression profiles. Pvyl-GR in Base was set to a relative intensity of 1. The mean ± SEM were calculated and displayed for *n* = 3 (2 biological, 1 technical) replicates. Pairwise *t*-tests with Welch’s correction were performed comparing Pvyl-PR or Pvyl-GR band intensities in low and high metabolite concentrations using Prism9 (*p* < 0.05 is significant).

### 4.17 Observation of PrdA activity in physiologically relevant conditions

This study uses clinical strains (Cd1, GCA_018885045.1; Cd3, GCA_018885005.1; cd5, GCA_018884945.1; cd8, GCA_018884905.1; cd11, GCA_018884845.1; cd17, GCA_018884725.1) isolated from subjects recruited at the Children’s Hospital of Pennsylvania (CHOP) from September 2015 to April 2018 (IRB approval number 15-011817), as previously described (Bushman et al., 2020). The work in our study did not require additional IRB approval as the strains are deidentified and contain no link to patient identifying information. No human patients were used in our study. We cite the initial study and include the IRB from that study which should be sufficient for our work. Here, all strains were cultivated anaerobically as described above in chemically defined medium (CDM) grown to stationary phase (OD_600_, of ∼0.8) or mid-log phase (OD_600_, of ∼0.4) (OD_600_ was detected by plate reader). CDM was prepared according to previously described recipe (Supplementary Table S5) (Cartman and Minton, 2010). In brief, fresh stock solutions of amino acids (5x), salts (10x), glucose (20x), trace salts (50x), iron (100x) and vitamins (100x) were made by dissolving components in dH_2_O and filter sterilizing. Appropriate amounts of each sterilized stock were then mixed with sterile dH_2_O. After treatment, cells were probe treated and subjected to fluorophore conjugation and gel-based analysis as described above. Band intensities of the active (probe-labelled) PrdA were quantified using ImageJ software (Schneider et al., 2012) and normalized against the corresponding expression profiles. The Pvyl-PR in *C. difficile* 630 was set to a relative intensity of 1. The mean ± SEM were calculated and displayed for *n* = 2 (biological) replicates. Multiple pairwise *t*-tests with Welch’s correction were performed comparing Pvyl-PR intensity in all strains using Prism9 (*p* < 0.05 is significant).

### 4.18 Fluorometric assay for D-proline reductase activity with hydrazine

D-proline reductase activity was assayed by the DTT- and D-proline reductase-dependent production of 5-aminovalerate adapted from previous study (Jackson et al., 2006). The reaction mixtures (250 μL) contained 100 mM potassium phosphate, pH = 8.0, 20 mM DTT, 10 mM MgCl_2_, 10 μg D-proline reductase (buffer transferred into 100 mM potassium phosphate, pH = 8.0, to remove glycerol) and 10 mM D-proline. Reactions (60 min, 30°C) were quenched with 3% trichloroacetic acid (100 μL of 10%). Following centrifugation to remove precipitated protein, 100 μL of reaction supernatant was placed into a 96-well microplate (Greiner bio-one), equilibrated to room temperature and 100 μL of *o*-phthalaldehyde mixture was added for detection of the fluorometric product of 5-aminovalerate and *o*-phthalaldehyde. The *o*-phthalaldehyde mixture was prepared fresh each time and contained 1 mL of *o*-phthalaldehyde (TCI) solution (1.192 M in 95% ethanol), 4 mL of 0.4 M boric acid buffer, pH 9.7, and 10 μL of 2-mercaptoethanol (EMD Millipore). Fluorescence was measured on a Tecan Infinite M1000 Pro at *λ*_ex_ = 340 nm, bandwidth = 5.0 nm and *λ*_em_ = 455 nm, bandwidth = 5.0 nm. Each reaction replicate was scanned three times and averaged. Each fluorescence reading from the 100 μL of reaction supernatant scanned was multiplied by 3.5 for total reaction fluorescence (250 μL reaction +100 μL trichloroacetic acid). Relative fluorescence was calculated from standard curves of 0–100 nmoles of 5-aminovalerate (Acros Organics, 153910050). The relative fluorescence was proportional to the amount of enzyme. The inhibition efficiency of organohydrazines **3**–**10** (see Materials) was assayed in 3-fold dilutions from 10.0 to 0.3 mM (**3**) or 3.0–0.1 mM (**4**–**10**). Only **4** and **7** had negligible fluorescence (<6% of D-proline reductase activity) at assay conditions. IC_50_ values and standard deviations were calculated from non-linear regression functions [log (inhibitor) vs. response] in Prism9 from three independent experiments.

## Data Availability

The original contributions presented in this study are publicly available. The mass spectrometry proteomics data was deposited to the ProteomeXchange Consortium *via* the PRIDE (Perez-Riverol et al., 2022) partner repository and can be found with dataset identifier PXD039143 and 10.6019/PXD039143.
