# Supplementary material for: Characterizing metabolic drivers of Clostridioides difficile infection with activity-based hydrazine probes
Source: Front Pharmacol. 2023 Jan 26;14:1074619. doi: 10.3389/fphar.2023.1074619 (PMC9908766; doi:10.3389/fphar.2023.1074619)
Supplement: Supplementary file 2 [file Presentation1.pdf]

Supplementary Material

1 Supplementary Tables and Figures

1.1 Supplementary Tables

Supplementary Table 1: Composition of minimal medium (MM)

Minimal Medium	*Component*	*Final Concentration (mg/L)*	*Final Concentration (mmol/L)*
Amino acids	Aspartate	100	1.0
	Glutamate	100	0.8
	Glycine	100	1.3
	Valine	100	0.9
	Proline	100	0.9
	Cysteine	100	0.8
	Phenylalanine	50	0.3
	Leucine	50	0.4
	Lysine	50	0.3
salts	Na_2HPO_4	5000	35.2
	KH_2PO_4	1000	7.3
	$MgSO_4$	100	0.8
	NaCl	2000	34.2
Glucose	*N*-acetylglucosamine	100	0.5
Iron	$FeSO_4 \cdot 7H_2O$	100	0.4
Vitamins	Nicotinic acid	1000	8.1
	Riboflavin	100	0.3

Supplementary Table 2: BHIS and MM sugar concentration

	mmol/L				
Solution	Test 1	Test 2	Test 3	Average	Standard Deviation
5 mM Glucose	1.166	1.166	1.221	1.2	0.03
15 mM Glucose	8.769	11.045	10.434	10.1	1.18
BHIS	15.429	14.097	14.430	14.7	0.69
MM	LO*	1.166	LO*	1.2	--

*Measurement range is 1.11 - 33.3 mmol/L

Supplementary Table 3: BHIS and MM amino acid quantitation

Amino acid	BHIS	MM
Hydroxyproline	6	
Histidine	326	
Asparagine	672	
Taurine	65	
Serine		
Glutamine	85	
Arginine	2164	31
Glycine	1698	2122
Aspartic Acid	21	829
Glutamic Acid	22	540
Threonine	1017	
Alanine	67	
GABA	35	
Proline	529	869
Hydroxylysine	82	
AABA	51	
Ornithine	73	
Cysteine		409
Lysine		359
Tyrosine	772	
Methionine	1281	
Valine		846
Isoleucine	1190	
Leucine		433
Phenylalanine		332
Tryptophan	1341	
**TOTAL (pmol/uL)**	**11497**	**6773**

Supplementary Table 4: Statistics of monoisotopic masses in MS1 spectra for chemically modified peptides

protein	*probe*	*media*	*captured peptide*	*modification*	*parent ion intensity*	*charge*	*$m/z_{theor.}$*	*average $m/z_{meas.}$*	*error (ppm)*	*SD*	*n*
PrdA	**1**	BHI	C*IGPASK	TEV_{heavy}	3.7E+07	2	542.8164	542.8160	-0.7	0.0004	279[†]
				TEV_{light}	4.5E+07	2	539.8095	539.8091	-0.6	0.0003	298[†]
		MM	C*IGPASK	TEV_{heavy}	5.5E+07	2	542.8164	542.8157	-1.2	0.0005	297[‡]
				TEV_{light}	6.7E+07	2	539.8095	539.8089	-1.1	0.0005	312[‡]
	**2**	BHI	C*IGPASK	TEV_{heavy}	7.2E+07	2	609.3427	609.3411	-2.7	0.0003	216[†]
				TEV_{light}	5.0E+07	2	606.3358	606.3351	-1.2	0.0006	107[†]
		MM	C*IGPASK	TEV_{heavy}	1.8E+08	2	609.3427	609.3409	-3.1	0.0003	223[§]
				TEV_{light}	1.7E+08	2	606.3358	606.3349	-1.6	0.0004	143[§]
GrdE	**1**	BHI	C*VSAC[‖]DKNPSYVHINNGVVEDLYAR	TEV_{heavy}	3.8E+06	4	808.9042	808.9036	-0.8	0.0008	72[†]
				TEV_{light}	3.8E+06	4	807.4007	807.4002	-0.9	0.0010	69[†]
		MM	C*VSAC[‖]DKNPSYVHINNGVVEDLYAR	TEV_{heavy}	4.4E+07	4	808.9042	808.9032	-1.2	0.0008	99[†]
				TEV_{light}	4.0E+07	4	807.4007	807.3996	-1.4	0.0006	104[†]
	**2**	BHI	C*VSAC[‖]DKNPSYVHINNGVVEDLYAR	TEV_{heavy}	4.3E+06	4	842.1674	842.1664	-1.2	0.0011	19[§]
				TEV_{light}	3.2E+06	4	840.6639	840.6640	-0.8	0.0013	18[§]
		MM	C*VSAC[‖]DK	TEV_{heavy}	8.0E+06	2	662.8266	662.8260	-0.9	0.0008	45[§]
				TEV_{light}	9.0E_06	2	659.8197	659.8187	-1.5	0.0006	46[§]

* site of modification by probe clicked with TEV tags.
[‖] site of static modification by carbamidomethyl.
[†] the sum of spectral counts ($n \geq 69$) for given peptide from three technical replicates (2 biological).
[‡] the sum of spectral counts ($n \geq 297$) for given peptide from four technical replicates (3 biological).
[§] the sum of spectral counts ($n \geq 18$) for given peptide from two biological replicates

Supplementary Table 5: Composition of chemically defined medium (CDM)

**Chemically Defined Medium**	***Component***	***Final Concentration (mg/L)***	***Final Concentration (mmol/L)***
Amino acids	casamino acids	10000	18.5
	L-tryptophan	500	2.4
	L-cysteine	500	4.1
Salts	Na_2HPO_4	5000	35.2
	$NaHCO_3$	5000	59.5
	KH_2PO_4	900	6.6
	NaCl	900	15.4
Glucose	D-glucose	10000	55.5
Trace salts	$(NH_4)_2SO_4$	40	0.3
	$CaCl_2•2H_2O$	26	0.2
	$MgCl_2•6H_2O$	20	0.1
	$MnCl_2•4H_2O$	10	0.05
	$CoCl_2•6H_2O$	1	0.008
Iron	$FeSO_4•7H_2O$	4	0.01
Vitamins	D-biotin	1	0.004
	calcium-D-pantothenate	1	0.004
	pyridoxine	1	0.006

1.2 Supplementary Figures

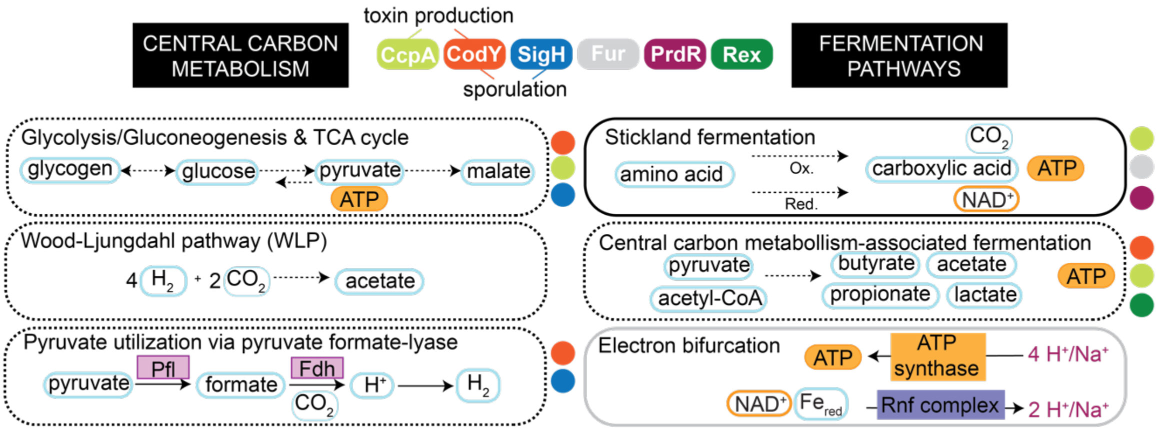

Supplementary Figure 1. Metabolic processes utilized by *C. difficile* and their association to toxin regulation. Simplified schematics of the metabolic pathways, both *Clostridia*-specific (outlined in full black) and not *Clostridia*-specific (outlined in dashed black), utilized by *C. difficile*. Electron bifurcation (outlined in gray) is coupled to multiple fermentation pathways. Dashed arrows represent multiple reactions. Global regulators (CcpA, CodY, SigH, Fur, PrdR, Rex) regulate the various pathways as marked by colored dots to the right of each pathway.

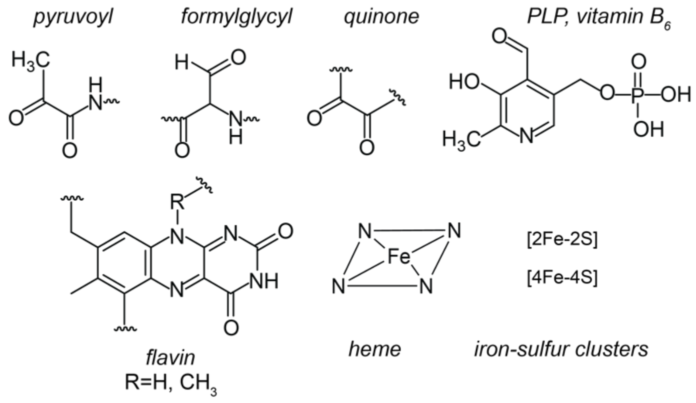

Supplementary Figure 2. Electrophilic and oxidative enzyme cofactors. Enzyme cofactors essential for bacteria.

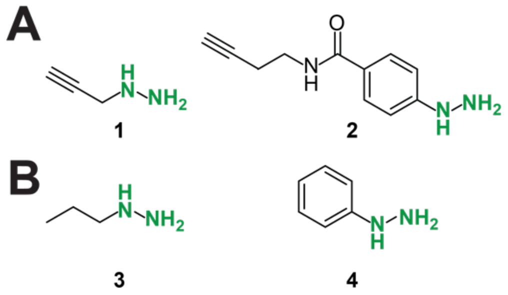

Supplementary Figure 3. Hydrazine probes and competitors. Structures of **(A)**, hydrazine probes and **(B)**, hydrazine competitors. Related to Fig. 1B.

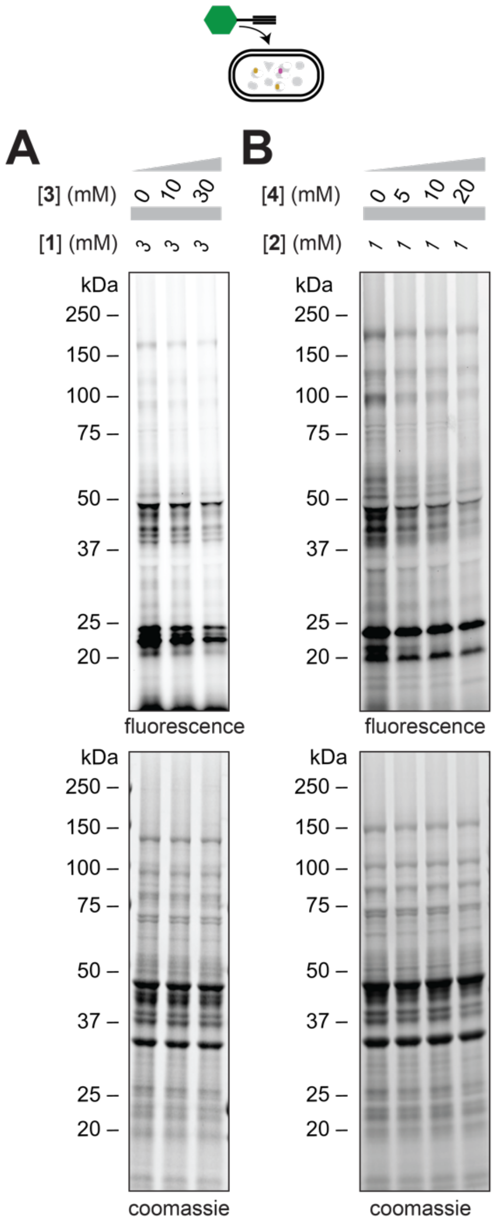

Supplementary Figure 4. Gel-based profiles of probe-treated *C. difficile* cells. **(A)**, Probe **1** and **(B)**, probe **2** labelling profiles in *C. difficile* cells cultivated in BHIS, grown to stationary phase and pretreated with varying concentrations of non-clickable analogs **3** and **4**, respectively (*upper*). Corresponding expression profiles are shown after Coomassie staining (*below*). Concentrations and molecular weight markers are indicated.

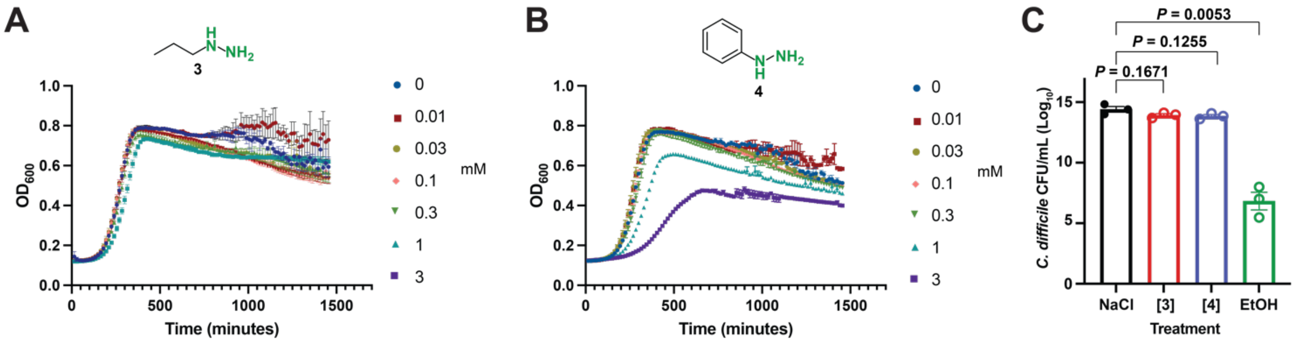

Supplementary Figure 5. *C. difficile* growth and viability in BHIS with hydrazine. Growth curves of *C. difficile* with 3-fold serial dilutions of **(A)**, **3** and **(B)**, **4** from 3.0-0.01 mM as assessed by optical density at 600 nm (OD_{600}). Hydrazine does not affect *C. difficile* growth at probe treatment conditions (3 mM for **1**, 1 mM for **2**). Averages of three independent experiments along with standard error are shown. **(C)**, Colony forming unit (CFU) of *C. difficile* with no treatment (NaCl, 3 mM), **3** (3 mM), **4** (1 mM) and Ethanol (20%). Hydrazine does not significantly affect *C. difficile* viability at probe treatment conditions (mean ± SEM, n=3, t-test with Welch's correction using Prism9, $P < 0.05$ is significant).

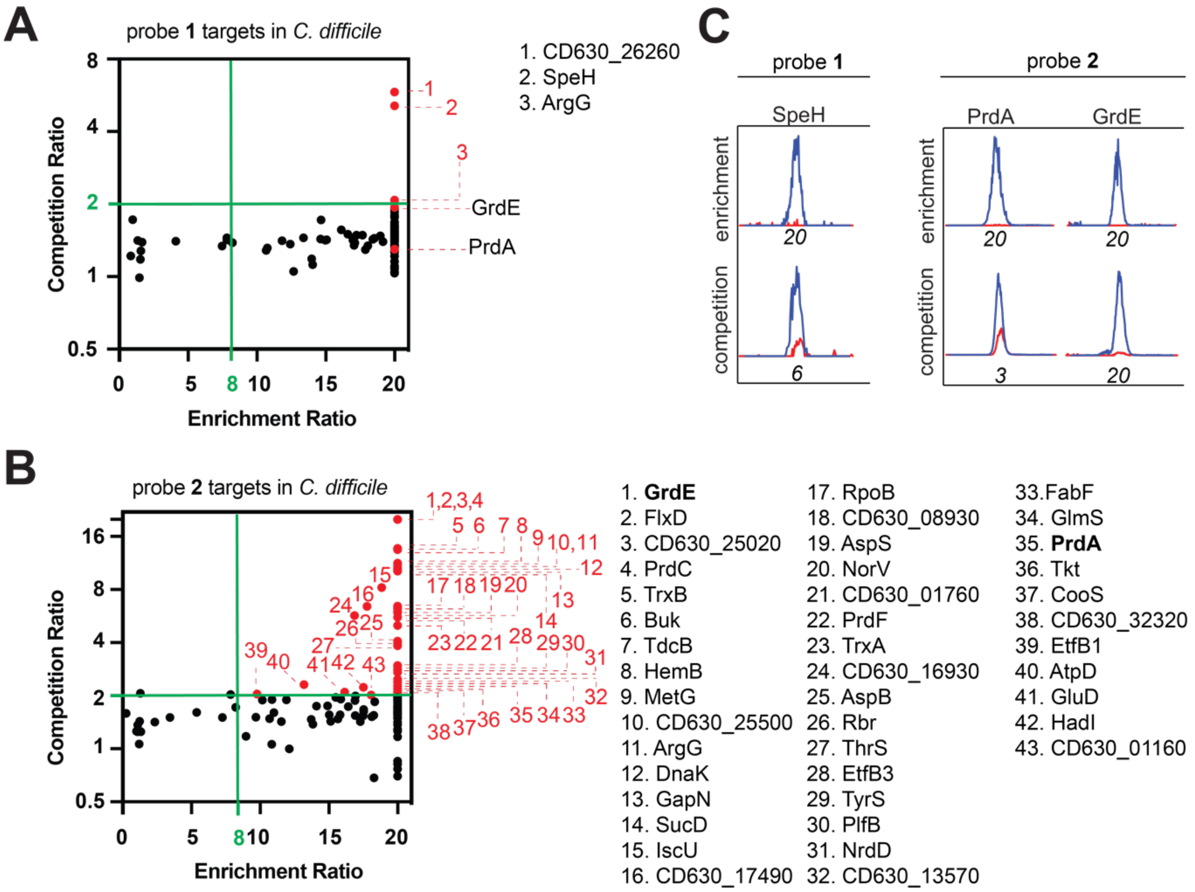

Supplementary Figure 6. Probe 1 and probe 2 reactive targets in *C. difficile* cells. Quadrant plot of average competition versus enrichment ReDiMe ratios for **(A)**, probe **1** and **(B)**, probe **2** from quantitative proteomic analysis of *C. difficile* VPI 10463 cells grown to stationary phase (*n* = 4 biological replicates). High-reactivity targets (enrichment ratio ≥ 8 and competition ratio ≥ 2) labelled by **1** or **2** are shown in the upper right quadrant in red and listed by protein symbol to the right of each plot. Enzymes further investigated in this work are in **bold**. **(C)**, Extracted MS1 chromatograms and corresponding heavy/light ratios (enrichment and competition) for representative tryptic peptides of endogenous forms of high-reactivity targets of probe **1** and probe **2**. Related to Fig 1.

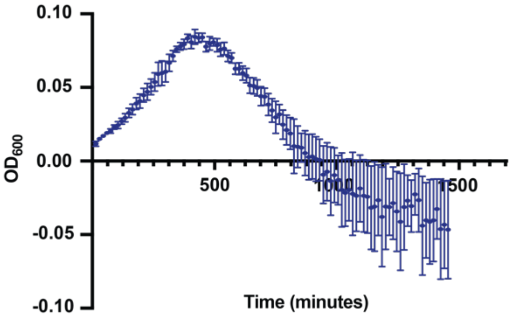

Supplementary Figure 7. Growth curve of *C. difficile* in MM. Growth curve of *C. difficile* cultivated in MM as assessed by optical density at 600 nm (OD_{600}). Averages of three independent experiments along with standard error are shown.

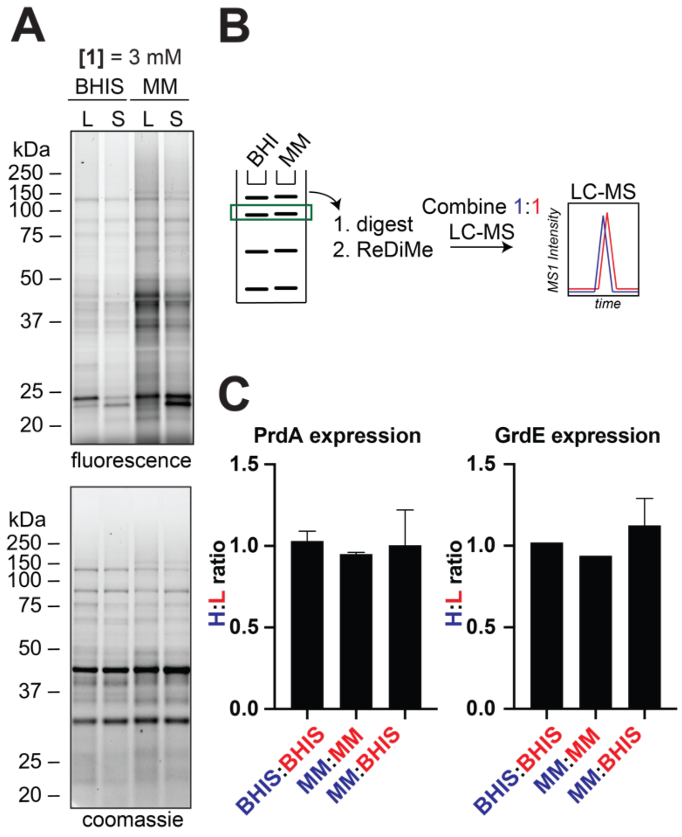

Supplementary Figure 8. Comparison of PrdA and GrdE expression in BHIS and MM. (A), Gel-based labelling profiles of probe **1** in the soluble proteome of *C. difficile* cells cultivated in BHIS and MM harvested in mid-log phase (L) and stationary phase (S) (*upper*). Corresponding expression profiles are shown after Coomassie staining (*below*). **(B)**, Schematic for in-gel digestion followed by late-stage reductive dimethylation (ReDiMe). **(C)**, Comparison of PrdA (*left*) and GrdE (*right*) expression in BHIS and MM in stationary phase after in-gel digestion and ReDiMe of the 20-25 kDa molecular weight range. H:L ratios quantify PrdA and GrdE expression in the various medias. Averages from two independent experiments along with standard deviations are shown. Related to Fig. 2.

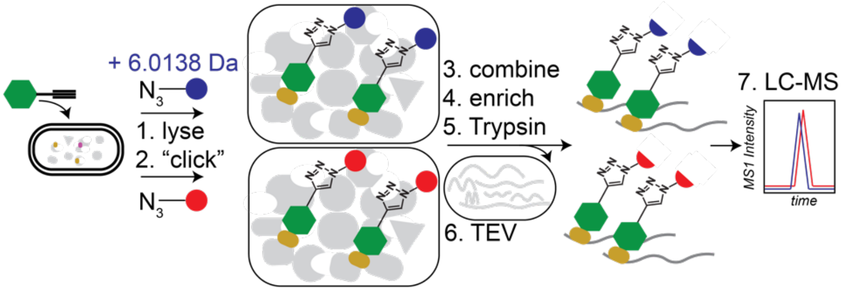

Supplementary Figure 9. Workflow schematic for site of labelling experiments. Characterization of probe-labelled peptides using the isoTOP-ABPP method. Probe-labelled peptides are conjugated to isotopically differentiated protease cleavable biotin-azide tags through 'click' chemistry, combined 1:1, enriched through streptavidin adsorption and digested with trypsin. The probe-labelled peptides are released from the beads through a second digestion with TEV protease and analyzed as mass differentiated pairs by LC-MS/MS.

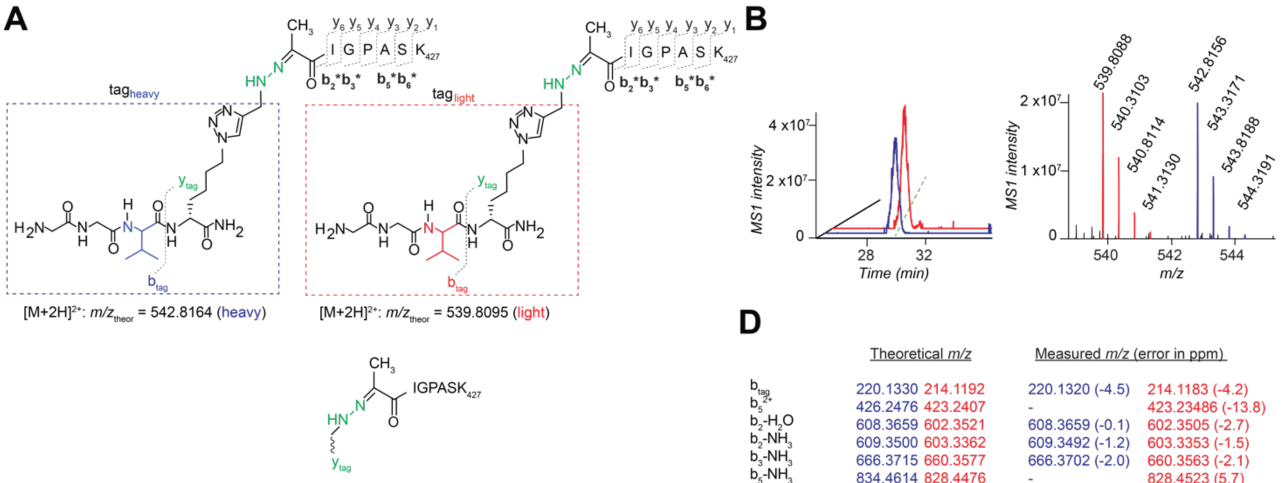

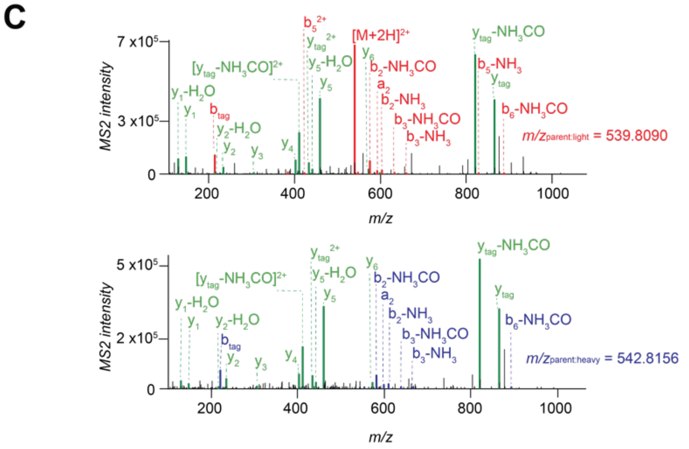

D

	Theoretical m/z		Measured m/z (error in ppm)	
b_{tag}	220.1330	214.1192	220.1320 (-4.5)	214.1183 (-4.2)
b_5^{2+}	426.2476	423.2407	-	423.23486 (-13.8)
b_2-H_2O	608.3659	602.3521	608.3659 (-0.1)	602.3505 (-2.7)
b_2-NH_3	609.3500	603.3362	609.3492 (-1.2)	603.3353 (-1.5)
b_3-NH_3	666.3715	660.3577	666.3702 (-2.0)	660.3563 (-2.1)
b_5-NH_3	834.4614	828.4476	-	828.4523 (5.7)
b_2-NH_3CO	581.3550	575.3412	581.3541 (-1.6)	575.3409 (-0.1)
b_3-NH_3CO	638.3765	632.3627	638.3724 (-6.4)	632.3604 (-3.8)
b_6-NH_3CO	893.4984	887.4846	893.4912 (-8.2)	887.4790 (-6.4)
$[b_2$-$NH_3]^{2+}$	305.1786	302.1717	305.1811 (8.1)	-
a_2	598.3816	592.3678	598.3807 (-1.5)	592.3662 (-2.7)
a_2-NH_3CO	553.3601	547.3463	553.3556 (-8.2)	547.3452 (-2.2)
a_3-NH_3CO	610.3816	604.3678	-	604.3639 (-6.5)
a_6-NH_3CO	865.5035	859.4897	865.4991 (-5.1)	-
y_6	572.3402		572.3398 (-0.6)	572.3403 (0.1)
y_5	459.2562		459.2557 (-1.1)	459.2558 (-0.9)
y_4	402.2347		402.2339 (-2.1)	402.2342 (-1.2)
y_3	305.1819		305.1811 (-2.5)	305.1808 (-3.7)
y_2	234.1448		234.1444 (-1.6)	234.1446 (-1.0)
y_1	147.1128		147.1125 (-2.0)	147.1126 (-1.1)
y_5-H_2O	441.2456		441.2450 (-1.4)	441.2453 (-0.8)
y_4-H_2O	384.2241		-	384.2239 (-0.6)
y_2-H_2O	216.1342		216.1341 (-0.8)	216.1341 (-0.5)
y_1-H_2O	129.1022		129.1020 (-1.6)	129.1022 (-0.3)
y_1-NH_3	130.0863		130.0860 (-2.2)	130.0862 (-0.5)
y_{tag}	865.4997		865.4991 (-0.7)	865.4991 (-0.7)
y_{tag}-NH_3CO	820.4782		820.4777 (-0.6)	820.4774 (-1.0)
y_{tag}^{2+}	433.2535		433.2534 (-0.2)	433.2533 (-0.5)
$[y_{tag}$-$NH_3CO]^{2+}$	410.7428		410.7426 (-0.5)	410.7427 (-0.2)

Supplementary Figure 10. MS characterization of probe 1-labelled peptide of PrdA in *C. difficile* cultivated in BHIS. **(A)**, Structures and theoretical parent masses of heavy- and light-tagged peptides processed by the isoTOP-ABPP method. **(B)**, Extracted parent ion chromatograms (*left*) and corresponding isotopic envelopes (*right*) for heavy- (blue) and light- (red) tagged peptides detected from *C. difficile* cells treated with probe **1** at stationary phase. **(C)**, MS2 spectra generated from parent ions. Unshifted ions are shown in green and the shifted ion series in blue (heavy) and red (light). **(D)**, Summary table of theoretical versus observed spectra assignments generated under high-resolution MS2 conditions.

A

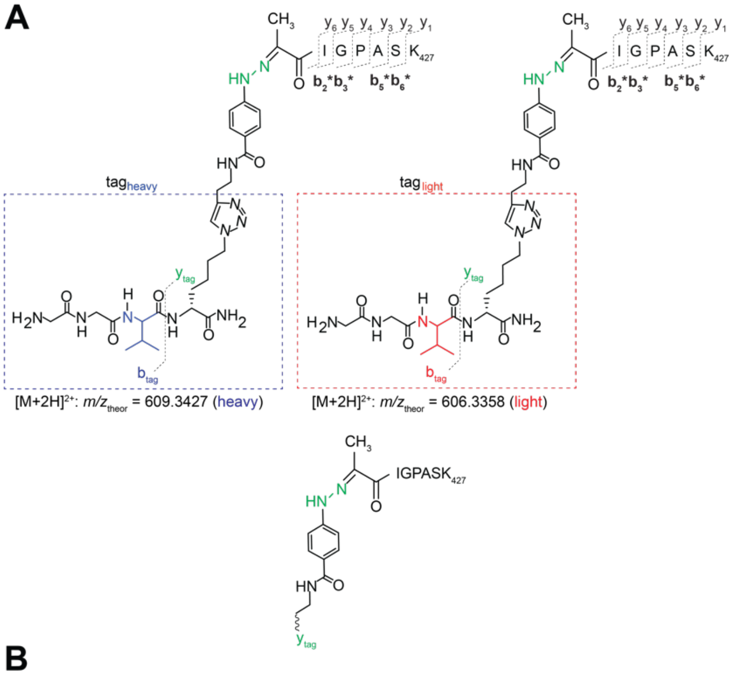

B

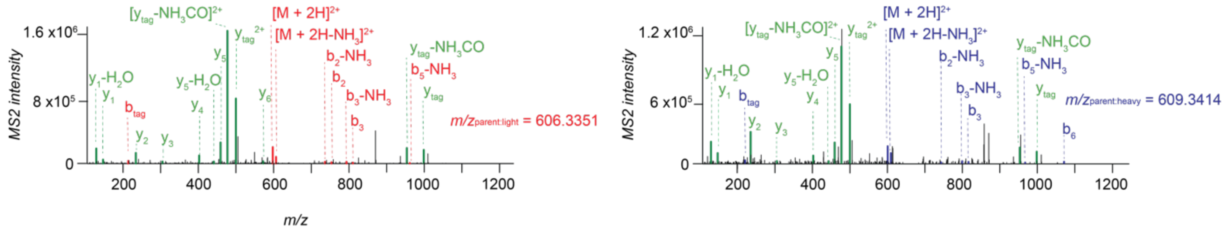

C

	Theoretical m/z		Measured m/z (error in ppm)	
b_{tag}	220.1330	214.1192	220.1322 (-3.6)	214.1184 (-3.7)
b_2	759.4293	753.4155	-	753.4145 (-1.3)
b_3	816.4507	810.4369	816.4477 (-3.7)	810.4356 (-1.6)
b_6	1071.5726	1065.5588	1071.5670(-5.2)	-
b_2-NH_3	742.4028	736.3890	742.4029 (0.1)	736.3893 (0.4)
b_3-NH_3	799.4242	793.4104	799.4226 (-2.0)	793.4106 (0.3)
b_5-NH_3	967.5141	961.5003	967.5198 (5.9)	961.5023 (2.1)
$[M + 2H]^{2+}$	609.3427	606.3358	609.3422 (-0.8)	606.3354 (-0.7)
$[M + 2H\text{-}NH_3]^{2+}$	600.8294	597.8225	600.8295 (0.2)	597.8223 (-0.3)
y_6	572.3402		-	572.3400 (-2.8)
y_5	459.2562		459.2562 (0.0)	459.2560 (-0.4)
y_4	402.2347		402.2346 (-0.2)	402.2344 (-0.7)
y_3	305.1819		305.1818 (-0.3)	305.1817 (-0.7)
y_2	234.1448		234.1447 (-0.4)	234.1445 (-1.3)
y_1	147.1128		147.1127 (-0.7)	147.1127 (-0.7)
y_5-H_2O	441.2456		441.2457 (0.2)	441.2454 (-0.5)
y_1-H_2O	129.1022		129.1022 (0.0)	129.1022 (0.0)
y_{tag}	998.5530		998.5513 (-1.7)	998.5515 (-1.5)
y_{tag}-NH_3CO	953.5316		953.5302 (-1.5)	953.5296 (-2.1)
y_{tag}^{2+}	499.7802		499.7801 (-0.2)	499.7799 (-0.6)
$[y_{tag}\text{-}NH_3CO]^{2+}$	477.2694		477.2693 (-0.2)	477.2691 (-0.6)

Supplementary Figure 11. MS characterization of probe 2-labelled peptide of PrdA in *C. difficile* cultivated in BHIS. **(A)**, Structures and theoretical parent masses of heavy- and light-tagged peptides processed by the isoTOP-ABPP method. **(B)**, MS2 spectra generated from parent ions. Unshifted ions are shown in green and the shifted ion series in blue (heavy) and red (light). **(C)**, Summary table of theoretical versus observed spectra assignments generated under high-resolution MS2 conditions. Related to Fig. 3.

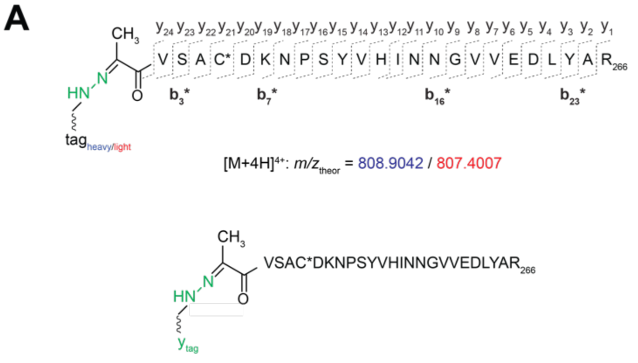

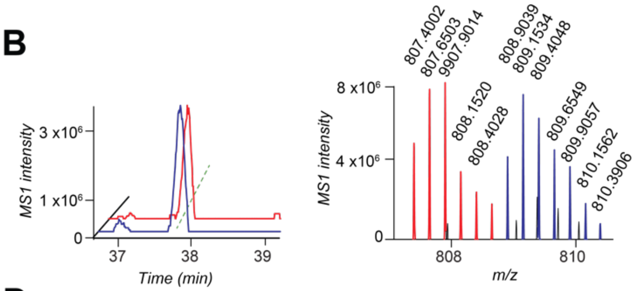

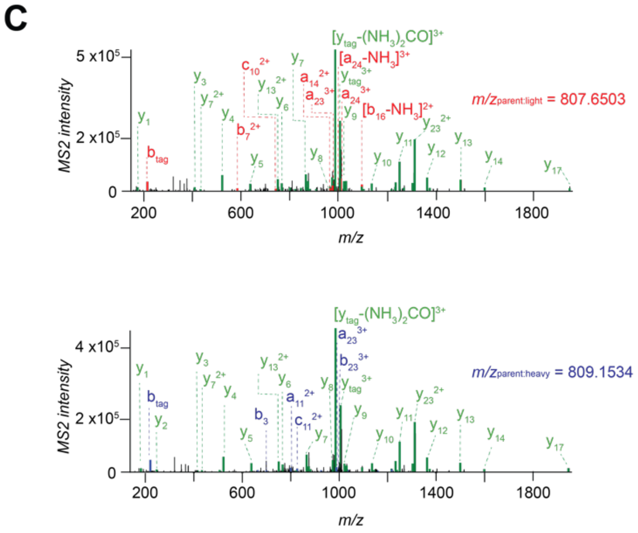

D

	Theoretical m/z		Measured m/z (error in ppm)	
b_{tag}	220.1330	214.1192	220.1323 (-3.2)	214.1184 (-3.7)
b_3	699.3934	693.3794	699.398 (6.6)	-
b_7^{2+}	587.2952	584.2882	-	584.2834 (-8.1)
b_{23}^{3+}	996.4870	994.4824	996.4951 (8.1)	-
$[b_{16}\text{-}NH_3]^{2+}$	1098.0260	1095.0636	-	1095.0636 (0.0)
a_{11}^{2+}	803.8932	800.8862	803.8868 (-7.9)	-
a_{14}^{2+}	978.4989	975.4919	-	975.4798 (-12.4)
a_{23}^{3+}	987.1554	985.1507	987.1477 (-7.8)	985.146545 (-4.2)
a_{24}^{3+}	1010.8344	1008.8298	-	1008.81604 (-13.6)
$[a_{24}\text{-}NH_3]^{3+}$	1515.7480	1003.1479	-	1003.1479 (0.0)
c_{10}^{2+}	744.8723	741.8653	-	741.8699 (6.2)
c_{11}^{2+}	826.4040	823.3970	826.3900 (-17.0)	-
y_{17}	1945.9767		1945.9816 (2.5)	1945.9763 (-0.2)
y_{14}	1598.8285		1598.8196 (-5.6)	1598.8226 (-3.7)
y_{13}	1499.7601		1499.7555 (-3.1)	1499.7554 (-3.2)
y_{12}	1362.7012		1362.6995 (-1.3)	1362.6987 (-1.8)
y_{11}	1249.6171		1249.6161 (-0.8)	1249.6160 (-0.9)
y_{10}	1135.5742		1135.5752 (0.9)	1135.5724 (-1.6)
y_9	1021.5313		1021.5289 (-2.3)	1021.5286 (-2.7)
y_8	964.5098		964.4994 (-10.8)	964.5016 (-8.5)
y_7	865.4414		865.4420 (0.6)	865.4425 (1.3)
y_6	766.3730		766.3725 (-0.7)	766.3722 (-1.1)
y_5	637.3304		637.3306 (0.3)	637.3297 (-1.2)
y_4	522.3035		522.3037 (0.4)	522.3032 (-0.6)
y_3	409.2194		409.2192 (-0.4)	409.2186 (-1.9)
y_2	246.1561		246.1557 (-1.8)	-
y_1	175.1190		175.1189 (-0.6)	175.1189 (-0.8)
$y_{15}\text{-}NH_3$	1232.5911		-	1232.5911 (0.0)
y_{23}^{2+}	1311.1243		1311.1234 (-0.7)	1311.1233 (-0.8)
y_{22}^{2+}	1267.6083		-	1267.6089 (0.5)
y_{21}^{2+}	1232.0897		1232.0918 (1.7)	1232.0885 (-1.0)
y_{20}^{2+}	1152.0744		1152.0739 (-0.5)	-
y_{19}^{2+}	1094.5609		1094.5568 (-3.8)	1094.5530 (-7.2)
y_{18}^{2+}	1030.5134		1030.5132 (-0.2)	1030.5107 (-2.6)
y_{17}^{2+}	973.4920		973.4903 (-1.8)	973.4904 (-1.7)
y_{16}^{2+}	924.9656		924.9628 (-3.1)	-
y_{15}^{2+}	881.4496		881.4503 (0.8)	881.4492 (-0.5)
y_{14}^{2+}	799.9179		799.9204 (3.1)	799.9181 (0.3)
y_{13}^{2+}	750.3837		750.3837 (-0.0)	750.3835 (-0.3)
y_7^{2+}	433.7585		433.2238 (-1.1)	433.2227 (-3.7)
$[y_{18}\text{-}NH_3]^{2+}$	1022.0047		-	1022.0047 (0.0)
y_{24}^{3+}	907.4414		-	907.4420 (0.6)
y_{23}^{3+}	874.4186		874.4175 (-1.3)	874.4168 (-2.0)
y_{22}^{3+}	845.4079		-	845.4099 (2.3)
y_{21}^{3+}	821.7289		821.7262 (-3.3)	821.7272 (-2.0)
y_{20}^{3+}	768.3853		-	768.3846 (-0.9)
y_{tag}^{3+}	1005.1612		1005.1615 (0.3)	1005.1609 (-0.3)
$[y_{tag}\text{-}(NH_3)_2CO]^{3+}$	984.4786		984.4789 (0.3)	984.4791 (0.5)

Supplementary Figure 12. MS characterization of probe 1-labelled peptide of GrdE in *C. difficile* cultivated in BHIS. **(A)**, Structures and theoretical parent masses of heavy- and light-tagged peptides processed by the isoTOP-ABPP method. C* is site of static modification by carbamidomethyl. **(B)**, Extracted parent ion chromatograms (*left*) and corresponding isotopic envelopes (*right*) for heavy- (blue) and light- (red) tagged peptides detected from *C. difficile* cells treated with probe **1** at stationary phase. **(C)**, MS2 spectra generated from parent ions. Unshifted ions are shown in green and the shifted ion series in blue (heavy) and red (light). **(D)**, Summary table of theoretical versus observed spectra assignments generated under high-resolution MS2 conditions.

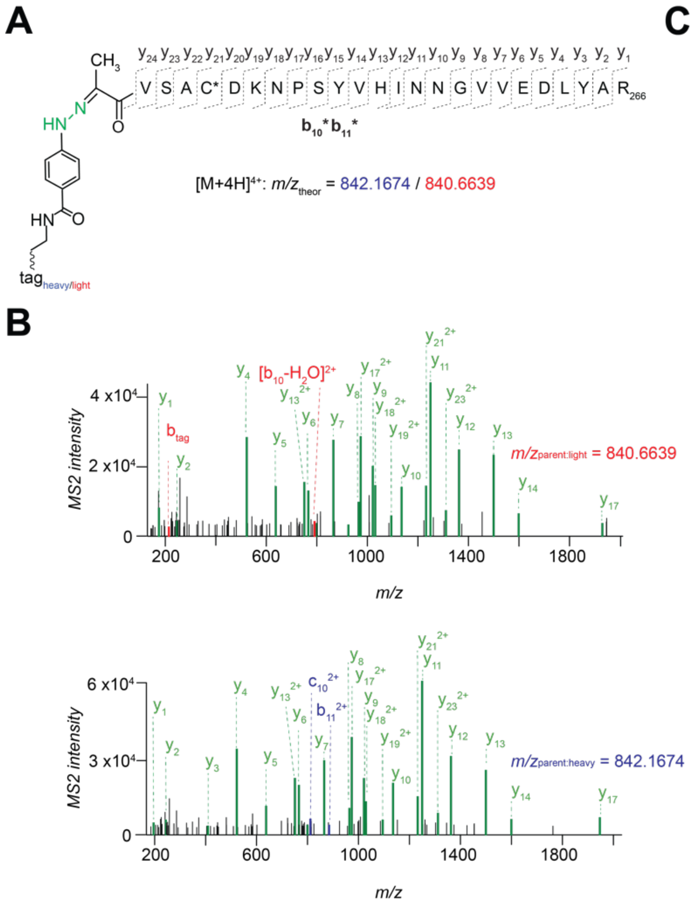

C

	Theoretical m/z		Measured m/z (error in ppm)	
b_{tag}	220.1330	214.1192	-	214.1180 (-5.6)
b_{11}^{2+}	884.4167	881.4097	884.4128 (-4.4)	-
$[b_{10}-H_2O]^{2+}$	793.8799	790.8730	-	790.8828 (12.4)
c_{10}^{2+}	811.3984	808.3915	811.4024 (4.9)	-
y_{17}	1945.9767		1945.9622 (-7.5)	1945.9443 (-16.6)
y_{14}	1598.8285		1598.8333 (3.0)	1598.8190 (-5.9)
y_{13}	1499.7601		1499.7555 (-3.1)	1499.7560 (-2.7)
y_{12}	1362.7012		1362.6986 (-1.9)	1362.6974 (-3.8)
y_{11}	1249.6171		1249.6160 (-0.9)	1249.6154 (-1.4)
y_{10}	1135.5742		1135.5721 (-1.8)	1135.5762 (1.8)
y_9	1021.5313		1021.5289 (-2.3)	1021.5261 (-2.8)
y_8	964.5098		964.5058 (-4.1)	964.5013 (-8.8)
y_7	865.4414		865.4409 (-0.6)	865.4405 (-1.0)
y_6	766.3730		766.3724 (-0.8)	766.3732 (0.3)
y_5	637.3304		637.3312 (1.3)	637.3300 (-0.6)
y_4	522.3035		522.3032 (-0.6)	522.3032 (-0.6)
y_3	409.2194		409.2193 (-0.2)	-
y_2	246.1561		246.1562 (0.4)	246.1563 (0.8)
y_1	175.1190		175.1187 (-1.7)	175.1189 (-0.6)
$y_{11}-NH_3$	1232.5889		1232.5889 (0.0)	-
y_{23}^{2+}	1311.1243		1311.1276 (2.5)	1311.1234 (-0.7)
y_{21}^{2+}	1232.0897		-	1232.0870 (-2.2)
y_{19}^{2+}	1094.5609		1094.5704 (8.7)	1094.5590 (-1.7)
y_{18}^{2+}	1030.5134		1030.5107 (-2.6)	1030.5128 (-0.6)
y_{17}^{2+}	973.4920		973.4913 (-0.7)	973.4921 (0.1)
y_{16}^{2+}	924.9656		-	924.9631 (-2.7)
y_{14}^{2+}	799.9179		799.9167 (-1.5)	799.9210 (3.9)
y_{13}^{2+}	750.3837		750.3831 (-0.8)	750.3833 (-0.5)

Supplementary Figure 13. MS characterization of probe 2-labelled peptide of GrdE in *C. difficile* cultivated in BHIS. **(A)**, Structures and theoretical parent masses of heavy- and light-tagged peptides processed by the isoTOP-ABPP method. C* is site of static modification by carbamidomethyl. **(B)**, MS2 spectra generated from parent ions. Unshifted ions are shown in green and the shifted ion series in blue (heavy) and red (light). **(C)**, Summary table of theoretical versus observed spectra assignments generated under high-resolution MS2 conditions. Related to Fig. 3.

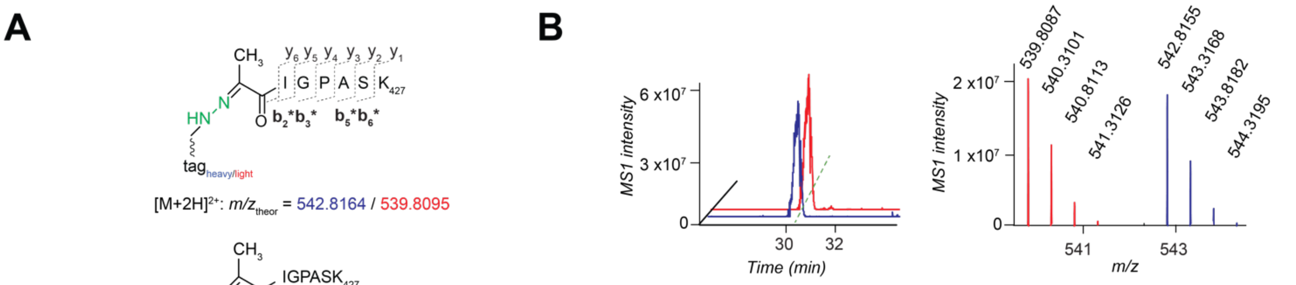

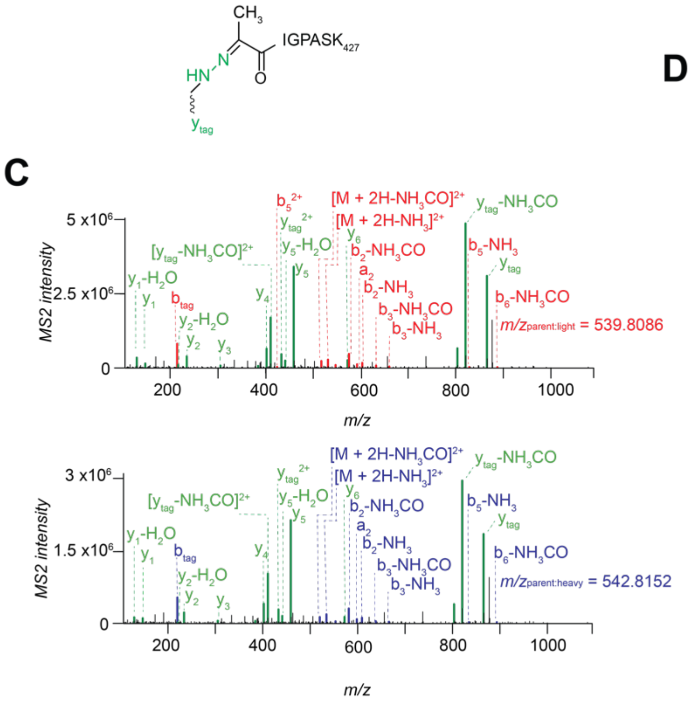

D

	Theoretical *m/z*		Measured *m/z* (error in ppm)	
b_{tag}	220.1330	214.1192	220.1322 (-3.6)	214.1184 (-3.7)
b_5^{2+}	426.2476	423.2407	-	423.2418 (2.6)
b_2-NH_3	609.3500	603.3362	609.3498 (-0.3)	603.3354 (-1.3)
b_3-NH_3	666.3715	660.3577	666.3690 (-3.8)	660.3577 (0.0)
b_5-NH_3	834.4614	828.4476	834.4630 (1.9)	828.4494 (2.2)
b_2-NH_3CO	581.3550	575.3412	581.3544 (-1.0)	575.3406 (-1.0)
b_3-NH_3CO	638.3765	632.3627	638.3740 (-3.9)	632.3618 (-1.4)
b_6-NH_3CO	893.4984	887.4846	893.4968 (-1.8)	887.4826 (-2.3)
$[b_2$-$NH_3]^{2+}$	305.1786	302.1717	305.1817 (10.2)	-
a_2	598.3816	592.3678	598.3805 (-1.8)	592.3673 (-2.8)
a_2-NH_3CO	553.3601	547.3463	553.3577 (-4.3)	547.3449 (-2.6)
$[M + 2H$-$NH_3]^{2+}$	534.3031	531.2962	534.3032 (0.2)	531.2958 (-0.8)
$[M + 2H$-$NH_3CO]^{2+}$	520.3056	517.2987	520.3049 (-1.3)	517.2978 (-1.7)
y_6	572.3402		572.3397 (-0.9)	572.3394 (-1.4)
y_5	459.2562		459.2559 (-0.7)	459.2558 (-1.4)
y_4	402.2347		402.2343 (-1.0)	402.2342 (-1.2)
y_3	305.1819		305.1817 (-0.7)	305.1819 (0.0)
y_2	234.1448		234.1447 (-0.4)	234.1446 (-0.9)
y_1	147.1128		147.1127(-0.7)	147.1127 (-0.7)
y_5-H_2O	441.2456		441.2455 (-0.2)	441.2454 (-0.5)
y_4-H_2O	384.2241		384.2255 (3.6)	384.2240 (-0.3)
y_2-H_2O	216.1342		216.1341 (-0.5)	216.1340 (-0.9)
y_1-H_2O	129.1022		129.1022 (0.0)	129.1022 (0.0)
y_1-NH_3	130.0863		130.0862 (-0.8)	130.0862 (-0.8)
y_{tag}	865.4997		865.4985 (-1.4)	865.4984 (-1.5)
y_{tag}-NH_3CO	820.4782		820.4773 (-1.1)	820.4773 (-1.1)
y_{tag}-$(NH_3)_2CO$	803.4595		803.4512 (-10.3)	803.4512 (-10.3)
y_{tag}^{2+}	433.2535		433.2537 (0.5)	433.2537 (0.5)
$[y_{tag}$-$NH_3CO]^{2+}$	410.7428		410.7429 (0.2)	410.7428 (0.0)

Supplementary Figure 14. MS characterization of probe 1-labelled peptide of PrdA in *C. difficile* cultivated in MM. **(A)**, Structures and theoretical parent masses of heavy- and light-tagged peptides processed by the isoTOP-ABPP method. **(B)**, Extracted parent ion chromatograms (*left*) and corresponding isotopic envelopes (*right*) for heavy- (blue) and light- (red) tagged peptides detected from *C. difficile* cells treated with probe **1** at stationary phase. **(C)**, MS2 spectra generated from parent ions. Unshifted ions are shown in green and the shifted ion series in blue (heavy) and red (light). **(D)**, Summary table of theoretical versus observed spectra assignments generated under high-resolution MS2 conditions.

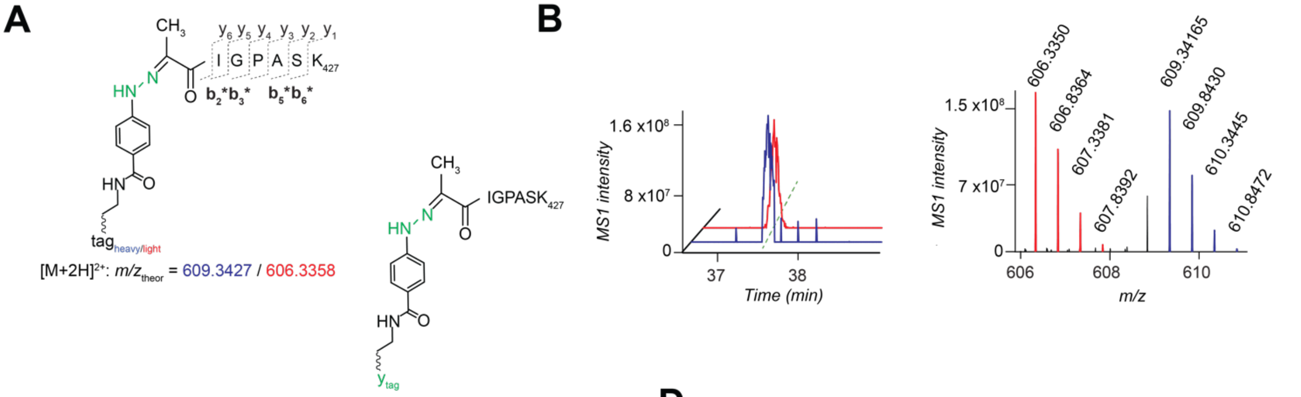

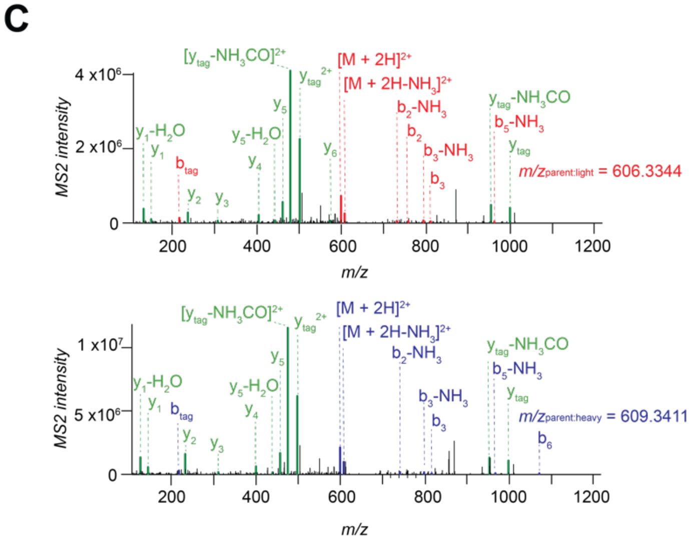

D

	Theoretical m/z		Measured m/z (error in ppm)	
b_{tag}	220.1330	214.1192	220.1321 (-4.1)	214.1182 (-4.7)
b_2	759.4293	753.4155	-	753.4146 (-1.2)
b_3	816.4507	810.4369	816.4467 (-4.9)	810.4360 (-1.1)
b_6	1071.5726	1065.5588	1071.5713 (-1.2)	-
b_2-NH_3	742.4028	736.3890	742.4028 (0.0)	736.3880 (-1.4)
b_3-NH_3	799.4242	793.4104	799.4215 (-3.4)	793.4083 (-2.6)
b_5-NH_3	967.5141	961.5003	967.5099 (-4.3)	961.4965 (-4.0)
$[M + 2H]^{2+}$	609.3427	606.3358	609.3414 (-2.1)	606.3352 (-1.0)
$[M + 2H\text{-}NH_3]^{2+}$	600.8294	597.8225	600.8285 (-1.5)	597.8220 (-2.8)
y_6	572.3402		-	572.3388 (-2.4)
y_5	459.2562		459.2555 (-1.5)	459.2558 (-0.9)
y_4	402.2347		402.2342 (-1.2)	402.2343 (-1.0)
y_3	305.1819		305.1815 (-1.3)	305.1815 (-1.3)
y_2	234.1448		234.1445 (-1.3)	234.1445 (-1.3)
y_1	147.1128		147.1125 (-2.0)	147.1125 (-2.0)
y_5-H_2O	441.2456		441.2451 (-1.1)	441.2454 (-0.5)
y_1-H_2O	129.1022		129.1021 (-0.8)	129.1021 (-0.8)
y_{tag}	998.5530		998.5499 (-3.1)	998.5512 (-1.8)
y_{tag}-NH_3CO	953.5316		953.5284 (-3.4)	953.5294 (-2.3)
y_{tag}^{2+}	499.7802		499.7794 (-1.6)	499.7798 (-0.8)
$[y_{tag}\text{-}NH_3CO]^{2+}$	477.2694		477.2688 (-1.3)	477.2690 (-0.8)

Supplementary Figure 15. MS characterization of probe2-labelled peptide of PrdA in *C. difficile* cultivated in MM. **(A)**, Structures and theoretical parent masses of heavy- and light-tagged peptides processed by the isoTOP-ABPP method. **(B)**, Extracted parent ion chromatograms (*left*) and corresponding isotopic envelopes (*right*) for heavy- (blue) and light- (red) tagged peptides detected from *C. difficile* cells treated with probe **2** at stationary phase. **(C)**, MS2 spectra generated from parent ions. Unshifted ions are shown in green and the shifted ion series in blue (heavy) and red (light). **(D)**, Summary table of theoretical versus observed spectra assignments generated under high-resolution MS2 conditions.

A

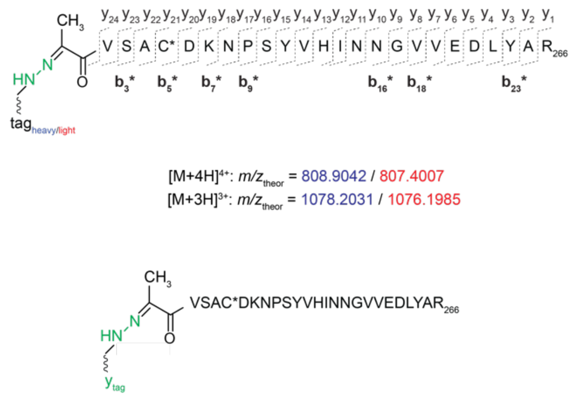

B

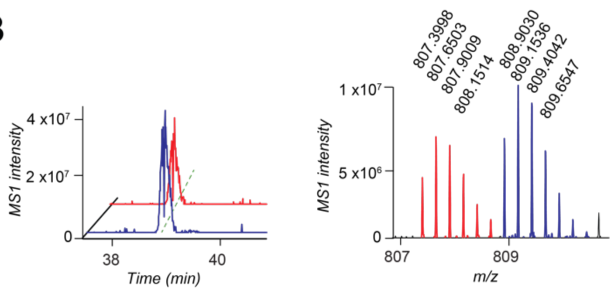

C

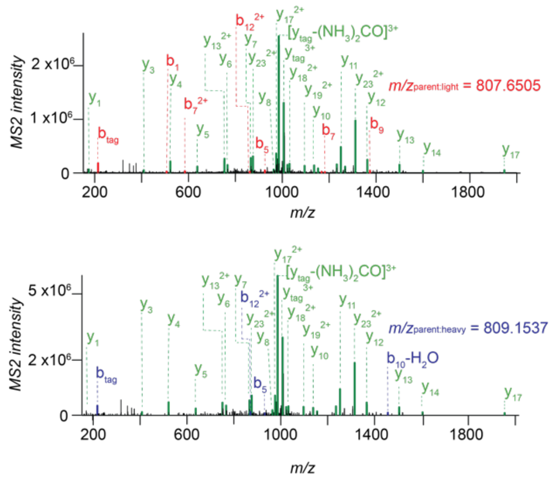

D

	Theoretical m/z		Measured m/z (error in ppm)	
b_{tag}	220.1330	214.1192	220.1322 (-3.6)	214.1185 (-3.3)
b_1	513.2929	507.2789	-	507.2845 (11.0)
b_3	699.3934	693.3794	-	-
b_5	930.4611	924.4471	930.4459 (-16.3)	924.4425 (-5.0)
b_7	1173.5830	1167.5690	-	1167.5734 (3.8)
b_9	1384.6787	1378.6647	-	1378.6571 (-5.5)
b_{10}-H_2O	1453.6675	1447.6861	1453.6675 (0.0)	-
b_7^{2+}	587.2952	584.2882	-	584.2842 (-6.8)
b_{12}^{2+}	867.4249	864.4179	867.4412 (18.8)	864.4180 (0.1)
b_{18}^{2+}	1184.5842	1181.5772	-	1181.5872 (-2.8)
b_{23}^{3+}	996.4870	994.4824	-	-
y_{17}	1945.9767		1945.9756 (-0.6)	1945.9773 (0.3)
y_{14}	1598.8285		1598.8259 (-1.6)	1598.8219 (-4.1)
y_{13}	1499.7601		1499.7556 (-3.0)	1499.7579 (-1.5)
y_{12}	1362.7012		1362.6987 (-1.8)	1362.7002 (-0.7)
y_{11}	1249.6171		1249.6160 (-0.9)	1249.6177 (0.5)
y_{10}	1135.5742		1135.5709 (-2.9)	1135.5737 (-0.4)
y_9	1021.5313		1021.5273 (-3.9)	1021.5294 (-1.9)
y_8	964.5098		964.5017 (-8.4)	964.5065 (-3.4)
y_7	865.4414		865.4401 (-1.5)	865.4409 (-0.6)
y_6	766.3730		766.3724 (-0.8)	766.3732 (0.3)
y_5	637.3304		637.3300 (-0.6)	637.3306 (0.3)
y_4	522.3035		522.3035 (0.0)	522.3037 (0.4)
y_3	409.2194		409.2182 (-2.9)	409.2197 (0.7)
y_2	246.1561		246.1555 (-2.4)	246.1561 (0.0)
y_1	175.1190		175.1189 (-0.6)	175.1188 (-1.1)
y_{23}^{2+}	1311.1243		1311.1226 (-1.3)	1311.1245 (0.2)
y_{22}^{2+}	1267.6083		1267.6049 (-2.7)	1267.6089 (0.5)
y_{21}^{2+}	1232.0897		1232.0907 (0.8)	1232.0906 (0.7)
y_{20}^{2+}	1152.0744		1152.0701 (-3.7)	1152.0739 (-0.4)
y_{19}^{2+}	1094.5609		1094.5575 (-3.1)	1094.5570 (-3.6)
y_{18}^{2+}	1030.5134		1030.5106 (-2.7)	1030.5120 (-1.4)
y_{17}^{2+}	973.4920		973.4891 (-3.0)	973.4907 (-1.3)
y_{16}^{2+}	924.9656		924.9630 (-2.8)	924.9589 (-7.2)
y_{15}^{2+}	881.4496		881.4491 (-0.6)	881.4489 (-0.8)
y_{14}^{2+}	799.9179		799.9169 (-1.3)	799.9185 (0.8)
y_{13}^{2+}	750.3837		750.3831 (-0.8)	750.3840 (0.4)
$[y_{18}\text{-}NH_3]^{2+}$	1022.0047		-	1022.0007 (-3.9)
y_{24}^{3+}	907.4414		-	907.4389 (-2.8)
y_{23}^{3+}	874.4186		874.4177(-1.0)	874.4189 (0.3)
y_{22}^{3+}	845.4079		-	845.4073 (-0.7)
y_{21}^{3+}	821.7289		-	821.7305 (1.9)
y_{20}^{3+}	768.3853		-	768.3786 (-8.7)
y_{tag}^{3+}	1005.1614		1005.1602 (-1.2)	1005.1615 (0.1)
$[y_{tag}\text{-}(NH_3)_2CO]^{3+}$	984.4788		984.4785 (-0.3)	984.4796 (0.8)

Supplementary Figure 16. MS characterization of probe 1-labelled peptide of GrdE in *C. difficile* cultivated in MM. **(A)**, Structures and theoretical parent masses of heavy- and light-tagged peptides processed by the isoTOP-ABPP method. C* is site of static modification by carbamidomethyl. **(B)**, Extracted parent ion chromatograms (*left*) and corresponding isotopic envelopes (*right*) for heavy- (blue) and light- (red) tagged peptides detected from *C. difficile* cells treated with probe **1** at stationary phase. **(C)**, MS2 spectra generated from parent ions. Unshifted ions are shown in green and the shifted ion series in blue (heavy) and red (light). **(D)**, Summary table of theoretical versus observed spectra assignments generated under high-resolution MS2 conditions.

A

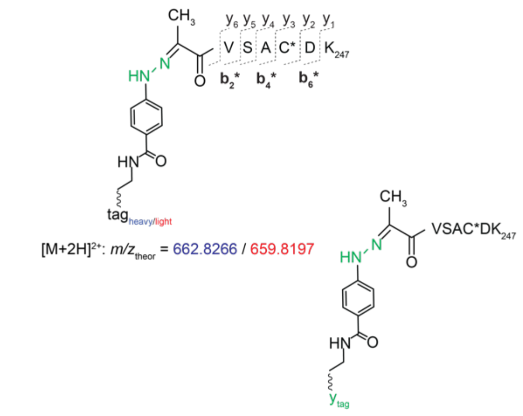

B

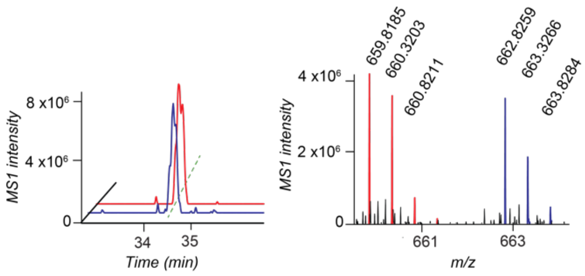

C

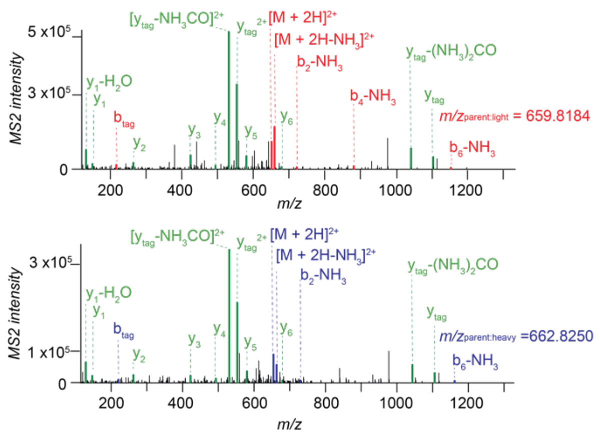

D

	Theoretical m/z		Measured m/z (error in ppm)	
b_{tag}	220.1330	214.1192	220.1318 (-5.5)	214.1181 (-5.1)
b_2-NH_3	728.3871	722.3733	728.3908 (5.1)	722.3724 (-1.2)
b_4-NH_3	886.4563	880.4425	-	880.4390 (-4.0)
b_6-NH_3	1161.5138	1155.5000	1161.4985 (-13.2)	1155.4874 (10.9)
$[M + 2H]^{2+}$	662.8266	659.8197	662.8269 (0.5)	659.8194 (-0.5)
$[M + 2H$-$NH_3]^{2+}$	654.3133	651.3064	654.3125 (-1.2)	651.3057 (-1.1)
y_6	679.3080		679.3044 (-5.3)	679.3044 (-5.3)
y_5	580.2395		580.2384 (-1.9)	580.2383 (-2.1)
y_4	493.2075		493.2058 (-3.4)	493.2070 (-2.8)
y_3	422.1704		422.1698 (-1.4)	422.1698 (-1.4)
y_2	262.1397		262.1391 (-2.3)	262.1390 (-2.7)
y_1	147.1128		147.1125 (-2.0)	147.1123 (-3.4)
y_1-H_2O	129.1022		129.1020 (-1.5)	129.1019 (-2.3)
y_{tag}	1105.5207		1105.5197 (-0.9)	1105.4874 (-30.1)
y_{tag}-$(NH_3)_2CO$	1043.4727		1043.4712 (-1.4)	1043.4703 (-2.3)
y_{tag}^{2+}	553.2640		553.2629 (-2.0)	553.2630 (-1.8)
$[y_{tag}$-$NH_3CO]^{2+}$	530.7533		530.7524 (-1.7)	530.7524 (-1.7)

Supplementary Figure 17. MS characterization of probe 2-labelled peptide of GrdE in *C. difficile* cultivated in MM. **(A)**, Structures and theoretical parent masses of heavy- and light-tagged peptides processed by the isoTOP-ABPP method. C* is site of static modification by carbamidomethyl. **(B)**, Extracted parent ion chromatograms (*left*) and corresponding isotopic envelopes (*right*) for heavy- (blue) and light- (red) tagged peptides detected from *C. difficile* cells treated with probe **2** at stationary phase. **(C)**, MS2 spectra generated from parent ions. Unshifted ions are shown in green and the shifted ion series in blue (heavy) and red (light). **(D)**, Summary table of theoretical versus observed spectra assignments generated under high-resolution MS2 conditions.

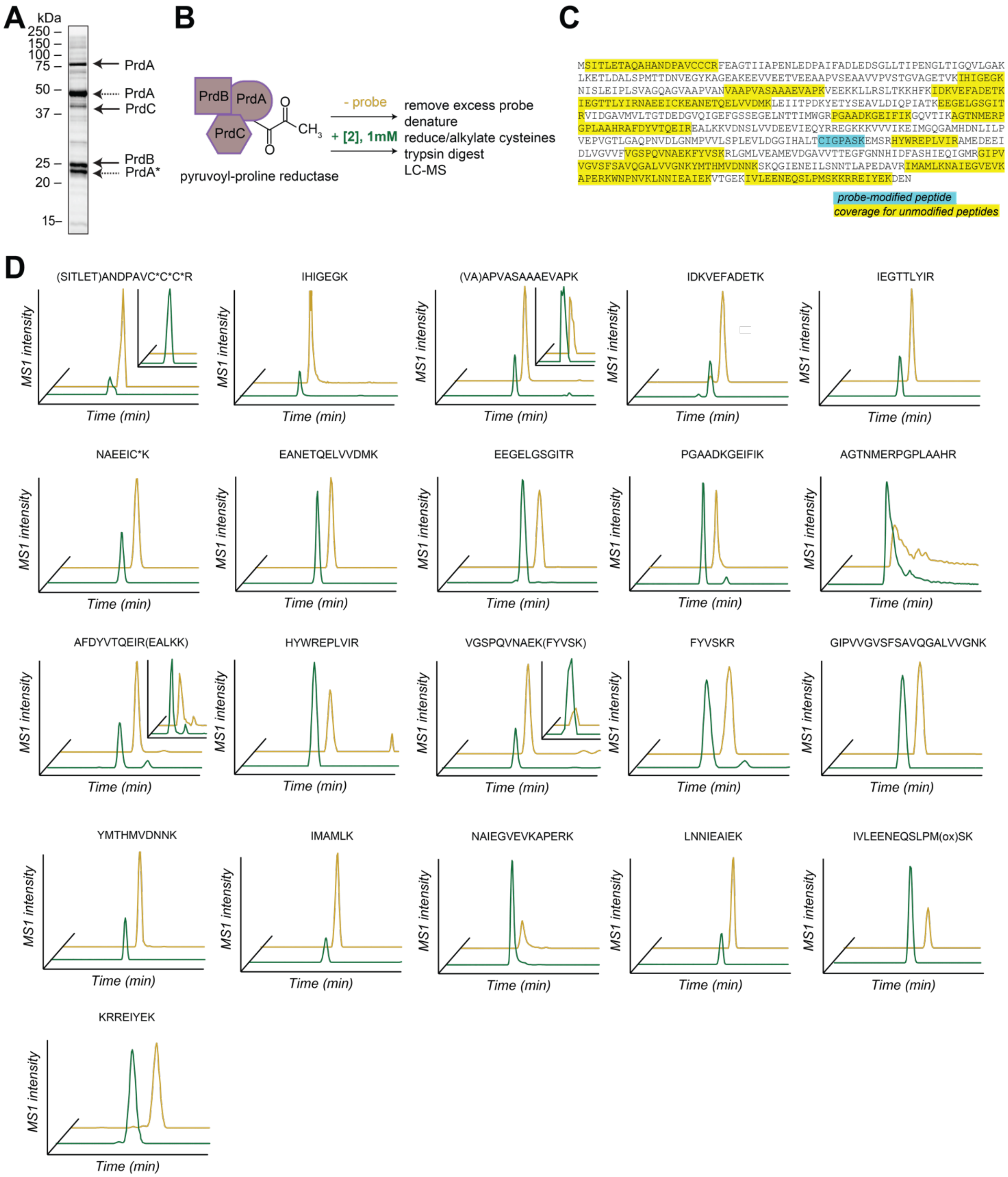

Supplementary Figure 18. Purification of PR and *in vitro* reactivity with probe 2. (A), Expression of purified PR from *C. difficile*. Subunits of the purified enzyme complex are labelled. **(B)**, Parallel samples of active D-proline reductase purified from *C. difficile* were incubated in the presence or absence of probe **2** (1 mM) and analyzed by LC-MS. **(C)**, Sequence coverage for unmodified and modified peptides of PrdA in D-proline reductase treated with probe **2**. **(D)**, Extracted parent ion

chromatograms for tryptic peptides highlighted in **(C)**. Inset chromatograms represent a different form of the tryptic peptide (shown in parentheses) observed from trypsin miscleavages. These are included to account for total unmodified peptide abundances. C* is site of static modification by carbamidomethyl. Related to Fig. 3.

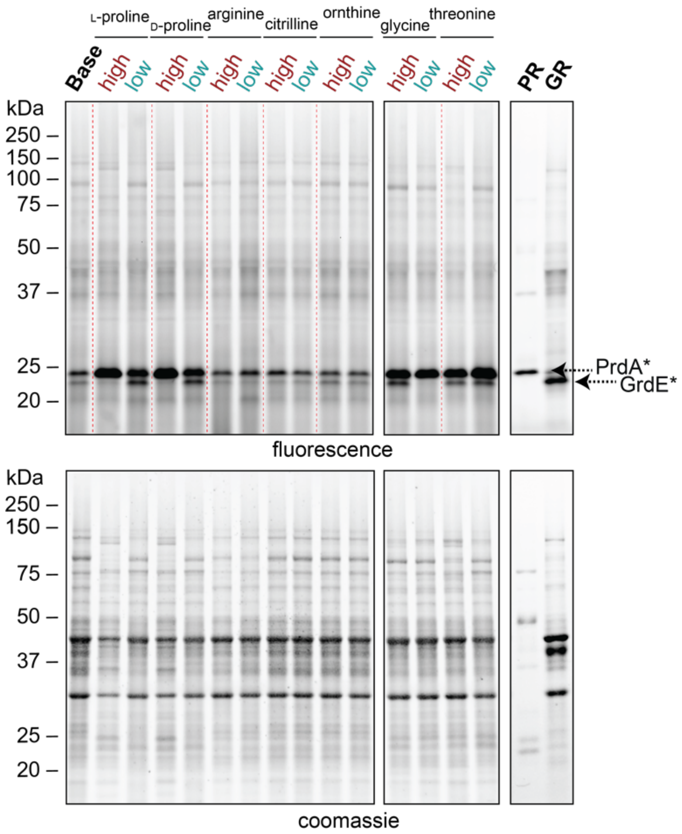

Supplementary Figure 19. 2-labelling and expression profiles of *C. difficile* in supplemented MM. Full gel-based monitoring of the fraction of Pvyl-PR and Pvyl-GR labelled by **2** in MM (Base) supplemented with high (10 mM) and low (0.1 mM) concentrations of selected metabolites (*upper*). **2**-labelled purified PR and **2**-treated lysate of glycine-grown cells are included as controls. Corresponding expression profiles are shown after Coomassie staining (*lower*). Related to Fig. 4.

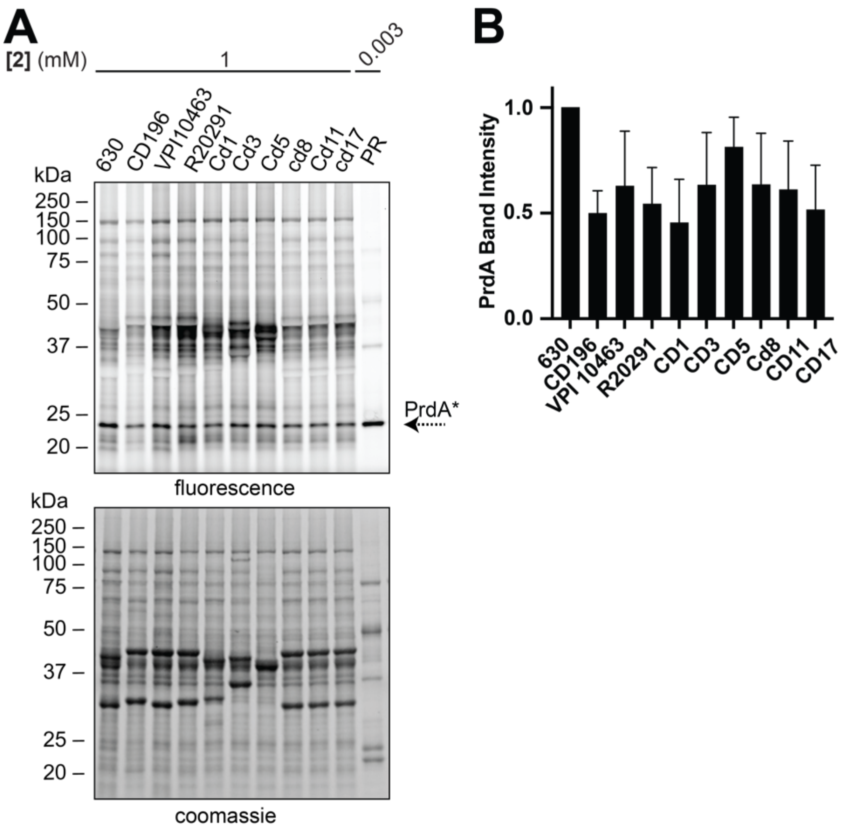

Supplementary Figure 20. Monitoring PR activity at stationary phase in physiologically relevant conditions. **(A)**, Gel-based proteomic profiles of *C. difficile* strains (in Fig. 5A) treated with **2** in stationary phase and the corresponding expression profiles after Coomassie staining (*lower*). **(B)**, Relative band intensities of Pvyl-PR in **(A)** normalized against expression. Pvyl-PR in strain 630 set to a relative intensity of 1. (mean ± SEM, n=2, multiple pairwise t-tests using Prism9, $P < 0.05$ is significant). Related to Fig. 5.

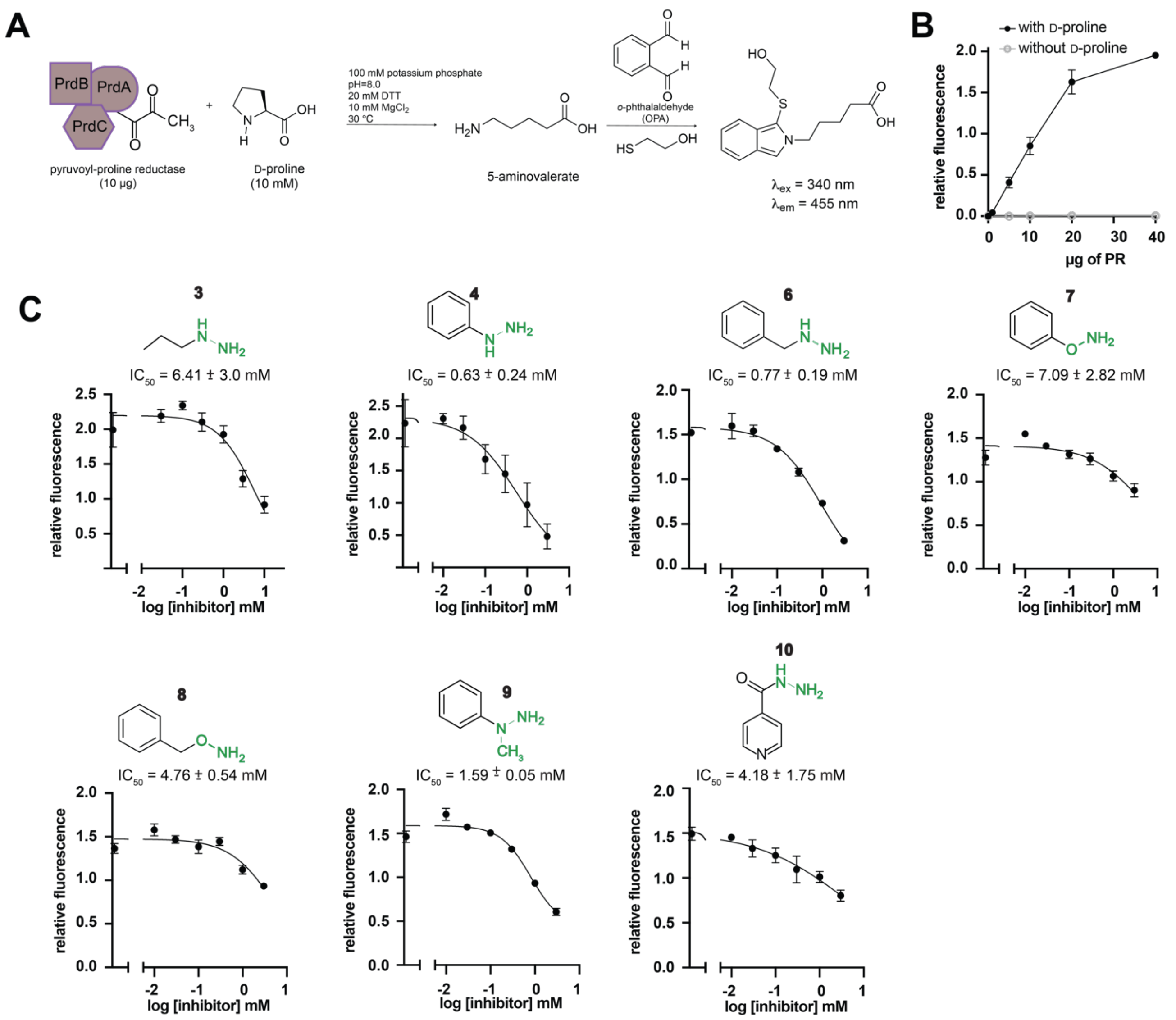

Supplementary Figure 21. Inhibition of D-proline reductase with organohydrazines. **(A)**, Schematic of fluorometric assay for measuring D-proline reductase activity. **(B)**, Purified PR activity in the absence and presence of D-proline. The relative fluorescence is proportional to the amount of 5-aminovalerate produced. Averages from three independent experiments along with standard error are shown. **(C)**, Inhibition curves of **3**, **4**, **6-10** for D-proline reductase. For all experiments, the relative fluorescence is proportional to the amount of 5-aminovalerate produced. Averages from three independent experiments along with standard error are shown and fitted to a nonlinear regression function (log[inhibitor] vs. response) in Prism 9. IC_{50} values and standard deviations were calculated from nonlinear regression functions from three independent experiments. Related to Fig. 5.
